# Cost-effectiveness of targeted feedback interventions after depression screening in primary care: health economic evaluation of the GET.FEEDBACK.GP trial

**DOI:** 10.1192/bjo.2025.10945

**Published:** 2026-02-02

**Authors:** Léon G. Kreis, Hans-Helmut König, Sebastian Kohlmann, Bernd Löwe, Martin Scherer, Christian Brettschneider

**Affiliations:** Department of Health Economics and Health Services Research, University Medical Center Hamburg-Eppendorf, Hamburg, Germany; Department of Psychosomatic Medicine and Psychotherapy, University Medical Center Hamburg-Eppendorf, Hamburg, Germany; Department of General Internal Medicine and Psychosomatics, Heidelberg University Hospital, Heidelberg, Germany; Institute of Primary Medical Care, University Medical Center Hamburg-Eppendorf, Hamburg, Germany

**Keywords:** Depression screening, cost-effectiveness, health economic evaluation, feedback intervention, primary care

## Abstract

**Background:**

Depression screening in primary care has been widely discussed, but its economic implications have remained largely unexplored. The GET.FEEDBACK.GP randomised controlled trial evaluated feedback interventions after depression screening in primary care. The study arms were (a) feedback provided to the general practitioner; (b) feedback to both the patient and the treating general practitioner; and (c) a control group without feedback. Analysis of clinical effectiveness revealed that feedback interventions were not associated with decreased depression severity. Their economic implications were the subject of this study.

**Aims:**

To evaluate the economic impact of general-practitioner- and patient-targeted feedback following depression screening for adults in German primary care.

**Method:**

A cost-effectiveness analysis from a societal perspective of feedback interventions after depression screening with a time horizon of 12 months was conducted. Direct and indirect costs were estimated. Quality-adjusted life years were calculated on the basis of the EQ-5D-5L, and incremental cost-effectiveness ratios and cost-effectiveness acceptability curves based on the net monetary benefit were constructed. Sensitivity analyses and *post hoc* explorative subpopulation analyses were performed. Trial registration: ClinicalTrials.gov, NCT03988985.

**Results:**

In total, 987 participants who screened positive for at least moderate depression were included. Feedback provision was not significantly associated with changes in costs or quality-adjusted life years during follow-up. Cost-effectiveness probabilities of feedback interventions were lower than 50% compared with no feedback. Higher cost-effectiveness probabilities were observed in patients whose suspected depression was confirmed 1 month post-screening and in those with previous depression.

**Conclusions:**

The analysed feedback interventions cannot be considered to be cost-effective for the investigated population. Patient-targeted feedback was potentially cost-effective for subpopulations, particularly patients with a later confirmed depression diagnosis; this requires further research.

Approximately 15% of individuals in Germany suffer from depression in their lifetime.^1^ As of 2019, depressive disorders were the 12th leading cause of disability-adjusted life years globally.^2^ Since the outbreak of the COVID-19 pandemic, an increase of 27.6% in new cases of major depressive disorder in 2020 was observed.^3^ Depressive disorders are highly comorbid with other mental and somatic diseases. Typical symptoms include lowered mood, reduced motivation, loss of pleasure or interest, and feelings of guilt.^4^ In addition, depression places a significant economic burden on society and is an important cause of sick leave and high excess costs.^5–7^

Depression goes undetected in primary care in about 50% of cases, and approximately 60% of depressed patients do not receive adequate specialised care.^8,9^ Screening for depression in primary care without additional staff-assisted care has been estimated not to improve depression outcomes.^10,11^ Systematic screening can be conducted on the basis of depression risk factors (such as previous depressive disorders or somatic risk factors) or as random screening in a general population. National guidelines on screening vary widely, ranging from recommending screening for all individuals (USA) to only high-risk groups (Germany, Canada) and not recommending screening at all (UK).^12–15^ There is also uncertainty regarding the optimal dissemination of screening results to effectively engage patients in a professional care process.

The literature on the health economic consequences of providing feedback in the form of diagnostic and therapeutic recommendations after depression screening is limited. Feedback provided to patients along with their treating physician for those with cardiac diseases has shown promising cost-effectiveness results, as suggested by the results of a randomised controlled trial (RCT). Cost-effectiveness probabilities were high (approximately 80%), independent of willingness-to-pay (WTP) margins.^16^ The *DISCOVER* trial revealed that automated feedback after internet-based depression screening had no significant effect on reductions in depressive symptoms.^17^ Its forthcoming health economic evaluation will provide further insights into the cost-effectiveness of online feedback interventions following depression screening.^18^

The three-armed multicentre RCT GET.FEEDBACK.GP evaluated patient-targeted feedback and feedback targeted at general practitioners (GPs) after depression screening in primary care.^19^ No significant reduction in depression severity due to the feedback interventions was observed.^20^ Nevertheless, the efficacy of feedback interventions in improved disease management was demonstrated in subgroups, especially women, those with previous depression, and those without any behavioural or substance addiction.^20^ Hence, a health economic analysis could offer important insights into the potential of (patient-targeted) feedback after depression screening to guide resource allocation.

The aim of this work was to evaluate the cost-effectiveness of GP-targeted and patient-targeted feedback after depression screening in the GET.FEEDBACK.GP trial. It represents the first cost-effectiveness analysis focusing on depression screening feedback interventions in German primary care.

## Method

### Study design

GET.FEEDBACK.GP was a three-armed multicentre RCT evaluating feedback interventions targeted at GPs and patients after depression screening in primary care.^19^ The trial was conducted in 64 general practices across five study centres, one in north Germany (Hamburg), one in east Germany (Jena), and three in south Germany (Munich, Tübingen, Heidelberg), and had a follow-up period of 12 months.

The authors assert that all procedures contributing to this work comply with the ethical standards of the relevant national and institutional committees on human experimentation and with the Helsinki Declaration of 1975, as revised in 2013. All procedures involving human participants and/or patients were approved by the ethics committee of the Medical Chamber (Hamburg, Germany) on 8 Apr 2019 (PV6031).^20^ The health economic evaluation was prespecified as part of the statistical analysis plan of GET.FEEDBACK.GP (available on ClinicalTrials.gov) and is reported on the basis of the Consolidated Health Economic Evaluation Reporting Standards (CHEERS) 2022.^21^ A completed version of the CHEERS 2022 checklist is provided as Supplementary Material 1 available at https://doi.org/10.1192/bjo.2025.10945.

### Participants

Patients were recruited and screened for depression in the waiting room before their GP appointment, regardless of the reason for their GP visit.^19^ Adult participants were randomly assigned to one of three study arms (1:1:1) if they scored 10 or higher on the nine-item Patient Health Questionnaire (PHQ-9), indicating at least moderate depressive symptoms. Patients were excluded if they indicated acute suicidality at baseline or reported having received a depression diagnosis and/or treatment (either pharmacological treatment or psychotherapy) in the previous year. Study nurses were blinded regarding the study arm of participating patients.^19^ Participants were required to provide written and electronic informed consent to be included in the trial. For more details on the recruitment process, refer to the publication of the clinical effectiveness study.^20^

In this health economic evaluation, we *post hoc* excluded participants who withdrew their consent during the follow-up period, as well as those who indicated acute suicidality and received additional interventions. Further details on inclusion and exclusion of participants, as well as follow-up rates, can be found in the participant flow chart in Supplementary Material 2.

### Interventions

Feedback was provided in closed envelopes by the study staff to the GPs and study participants directly after depression screening. GPs and patients were given time to read the feedback before the consultation. Otherwise, no professional guidance by the study staff was provided to GPs or patients.

The trial consisted of three study arms. The no-feedback arm underwent depression screening without receiving any feedback or result. In this group, patients received a thank-you letter and GPs a note stating that the patient had participated in the trial. In the GP-targeted feedback arm, patients received a general thank-you letter, whereas treating GPs received the information that their patients had screened positive for depressive symptoms, a disclaimer that a depression diagnosis is not possible on the basis of screening alone, and recommendations on further assessment and treatment. In the GP-targeted plus patient-targeted feedback group, GPs received the same feedback as in the GP-targeted feedback group, and patients also received written feedback (including their screening results, guideline-based depression information, a recommendation to mention the screening result to their GP, and contact options). Further details of the interventions have been reported elsewhere.^19,20^

### Data collection and measures

#### Data collection

Data were collected at four time points. *T*_0_ was the baseline assessment, conducted between 17 July 2019 and 31 January 2022. *T*_1_, *T*_2_ and *T*_3_ represented follow-up after 1, 6 and 12 months, respectively. The study protocol provides detailed information on the data collection process.^19^

Depression severity was assessed using the PHQ-9,^22^ and health-related quality of life (HRQL) was measured using the EuroQol-5D-5L (EQ-5D-5L).^23^ Furthermore, depression diagnoses were ascertained using the Mini-International Neuropsychiatric Interview (MINI) at *T*_1_, *T*_2_ and *T*_3_.^24^ Healthcare use, including medication intake and productivity losses, was assessed by a modified version of the Client Sociodemographic and Service Receipt Inventory.^25^

#### EQ-5D-5L

The EQ-5D-5L is a questionnaire to assess HRQL in five dimensions: mobility, self-care, usual activities, pain and/or discomfort, and anxiety and/or depression.^23^ Respondents rate their HRQL in each dimension on a five-point scale ranging from 1 (no problems) to 5 (extreme problems), resulting in 5^5^ = 3125 different health states.

The EQ-5D-5L is broadly accepted and applied as an HRQL measure; its psychometric properties are considered to be reliable and have been well established.^26^ In the base case analysis, the German value set was applied to the EQ-5D-5L descriptive system, resulting in EQ-5D-5L index values ranging from −0.661 (worst imaginable health state) to 1 (best imaginable health state).^27^

#### PHQ-9

The PHQ-9 is a measure of depressive symptom severity that is often used as a screening instrument.^22,28^ It consists of nine questions regarding the frequency of typical depressive symptoms using a four-point scale (0–3). The overall PHQ-9 score is the sum of points per item. Depression severity is indicated by five levels: no/minimal (PHQ-9 score 0–4), mild (5–9), moderate (10–14), moderately severe (15–19) and severe (20–27) depression.^22^ The PHQ-9 is considered to have both high specificity and sensitivity, as well as superior psychometric characteristics.^29^

#### Healthcare service use and costs

Healthcare service use was estimated from participants’ responses to an adapted version of the Client Sociodemographic and Service Receipt Inventory throughout the trial.^25^ Data on in-patient, out-patient and non-physician services (physiotherapy, massage, etc.) and pharmaceutical intake, as well as formal and informal nursing care, were collected. At baseline, numbers of medications, GP visits, in-patient stays and sick leave days were assessed.

Direct and indirect costs were calculated on the basis of German standard unit costs.^30^ For medication costs, a German pharmaceutical directory (*Rote Liste*) including pharmacy retail prices was used.^31^ Informal care was estimated with the replacement cost approach, using gross labour costs for ‘social work (excluding homes)’ (economic sector Q88) plus non-wage labour costs as reference costs.^32,33^ Indirect costs included productivity losses due to absenteeism from paid work and were estimated using the human capital approach on the basis of gross labour costs in Germany.^34^ In this health economic evaluation, intervention costs were disregarded because differences in intervention costs between study arms consisted only of printing costs for one or two sheets of paper. Overall intervention costs consisting of technology and personnel costs (compared with no screening) were approximated on the basis of publicly available data. As these costs were minimal, they were disregarded in the analysis, but they are presented in Supplementary Material 3. All additional costs triggered by the intervention were covered in the costs of healthcare service use. Therefore, intervention costs were considered to be negligible and not relevant to decision-making from a societal perspective. Costs (€) were adjusted to the reference year of 2022 by applying the consumer price index.^35^ An overview of all considered unit costs is provided in Supplementary Material 4. Total costs were estimated by summation of costs across all available categories and time points.

#### QALY calculation

Quality-adjusted life years (QALYs) were estimated on the basis of EQ-5D-5L index scores.^36^ QALYs between consecutive time points were assumed to be subject to a linear trend and calculated as follows:

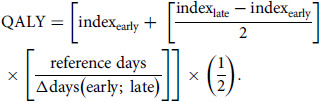




Total QALYs in the follow-up period were the sum of QALYs between consecutive follow-up surveys. Reference days were 30 for the period between *T*_0_ and *T*_1_ (1 month), 150 for the time between *T*_1_ and *T*_2_ (5 months), and 180 between *T*_2_ and *T*_3_ (6 months). Neither QALYs nor costs were discounted owing to the short follow-up period.

### Statistical analysis

Participants were analysed on the basis of the intention-to-treat principle. Owing to this approach, missing data were common. In the data-set used for the health economic evaluation, approximately 20% of values were missing. Hence, we created 20 complete data-sets by applying multiple imputation by chained equations, assuming data were missing at random (MAR).^37^ To assess this assumption, we performed descriptive and visual investigations of missingness patterns, as well as logistic regressions to analyse associations between baseline variables and missingness in outcome variables. Logistic regressions revealed that missingness of health economic outcomes (costs or QALYs) were predicted by being a non-native German speaker, smoking, lower HRQL (EQ-5D-5L index, PHQ-9 score) and a higher number of GP visits, as well as reporting depressive risk factors (addiction, persistent somatic symptoms, no social support). Hence, assumed that missingness depended on the observed values, which supported the MAR assumption. A report on missing values in the data-set is provided in Supplementary Material 5. All statistical analyses were conducted using R (version 4.4.2, https://www.r-project.org/) using RStudio for Windows.^38^

#### Base case analysis

In the base case analysis, we focused on the cost-effectiveness of the interventions from a societal perspective, considering a 1-year time horizon. Regarding costs, all available direct costs from healthcare service use, medication and informal care were included. Productivity losses due to absenteeism were incorporated as indirect costs. As effects, QALYs estimated using the EQ-5D-5L were used.

Before the analysis, we evaluated systematic differences between study arms using linear ordinary least squares regressions for continuous variables (age, baseline in-patient days, number of medications, GP visits, EQ-5D-5L index, EQ VAS, PHQ-9), binary logistic regressions for binary variables (health insurance plan, school education ≥10 years) and multinomial logistic regressions for non-binary categorical variables (gender, city size).

Cost differences during follow-up were estimated by applying generalised linear mixed models with a gamma distribution and a log-link function. Effect differences in QALYs were estimated using linear mixed-effects regression models. In both models, random effects for practices nested in study centres were assumed. As fixed effects, we considered a set of sociodemographic characteristics (age, gender, city size, living situation, education), depression risk factors (depression history, current and recent pregnancy), baseline PHQ-9 and EQ-5D-5L scores, and baseline GP visits and sick leave days as covariates. We assumed a significance level of 5%. Unadjusted incremental cost-effectiveness ratios (ICERs) between study arms were estimated to describe the additional cost per incremental unit of (health) effects due to an intervention. ICER was calculated as follows:

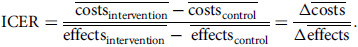




As the ICER is a point estimate and insensitive to data uncertainty, cost-effectiveness acceptability curves (CEACs) were constructed to visualise cost-effectiveness probabilities for different WTP thresholds.^39^ Net benefit regressions (NBRs) were conducted considering WTP margins between €0 and €160 000 per QALY with €10 000 increments. The detailed rationale behind these methods can be found elsewhere.^40–42^ Briefly, NBRs estimate the probability of cost-effectiveness, i.e. the likelihood that the intervention has a positive net benefit. This is done using the *P*-value of the group or intervention estimator in the NBR, with 1/2 the *P*-value used when the net benefit is negative and 1 − 1/2 the *P*-value when the net benefit of the intervention is positive. For the NBR, we again applied linear mixed-effects regression models using the covariates described above. All analyses were conducted pairwise, comparing each intervention group separately with the no-feedback group.

#### Deterministic sensitivity analyses

We performed sensitivity analyses for three scenarios. In the first scenario analysis, we focused on costs from a healthcare payer perspective, including all direct costs except informal care. In this scenario, absenteeism at baseline was excluded from the list of covariates. As the second deterministic sensitivity analysis, and to test the robustness of the multiple imputation approach, we performed a complete case analysis. In this scenario, only participants for whom both societal costs and QALYs for the 12-month follow-up were available (not missing) were included.

In the third scenario, we calculated depression-free days (DFDs) on the basis of PHQ-9 scores, estimating ICERs in €/DFD. We assumed a full DFD when the PHQ-9 score was below 5 (no or minimal depression). When the PHQ-9 score was 15 or higher (indicating moderately severe depression), we assumed no DFD. For PHQ-9 values between 5 and 14, we used linear interpolation to estimate the probability that a participant had a DFD. We assumed linear changes in PHQ-9 scores, analogous to the calculation of QALYs. This reliable and well-validated approach was consistent with that used in previously published studies.^43,44^ To determine a cost-effectiveness threshold per DFD, we adapted the approach used by Unützer et al^45^ to the German setting by weighting their threshold with the gross household income in Germany.^46^ Furthermore, we calculated a CEAC for WTP margins between €0/DFD and €200/DFD with €20/DFD increments.

#### *Post-hoc* explorative subpopulation analyses

In addition, we conducted *post hoc* explorative subpopulations analyses to further disentangle possible cost and effect differences and determine subpopulations for which the interventions could possibly be cost-effective. As these analyses were not pre-specified,^19^ they should be considered to be exploratory, and their results need to be handled with care. We focused on the categories for which the GP-targeted plus patient-targeted feedback intervention was associated with a significant (significance level: *P* = 0.1) decrease in the primary outcome of clinical effectiveness (depression severity), namely female gender, having had a previous depressive episode and the absence of any comorbid addiction.^20^ Therefore, we considered gender, depression history, and whether patients had any (behavioural or substance) addiction at baseline. We also estimated the costs, effects and cost-effectiveness of feedback interventions for the subpopulation of participants whose suspected depression was confirmed 1 month after baseline using the MINI diagnostic interview^24^ compared with those who had not been diagnosed.

We performed subpopulation analyses by dividing the imputed data-sets with respect to the categories of interest and then conducting all analyses as previously described. Hence, we estimated cost and effect differences between study arms for subpopulations as well as cost-effectiveness. We applied the same covariates as described previously, excluding covariates with too few observations per group (pregnancy, breastfeeding and living situation), and city size was transformed to a binary variable. As randomisation was not stratified considering these categories, the results of these analyses should be interpreted cautiously and seen as inspiration for further research.

## Results

### Characteristics of the study population

In total, 987 (no feedback: 329; GP-targeted feedback: 329; GP-targeted plus patient-targeted feedback: 329) participants were included in this analysis. Of these, 614 patients (62.2%) were female, and the mean age was 39.5 (s.d. = 15.3) years. The average depression severity at baseline was moderate (mean PHQ-9 score: 13.5, s.d. = 3.3), as reported by 69.5% of participants. The mean HRQL as measured by the EQ-5D-5L was 0.675 (s.d. = 0.261). Table 1 presents the differences between study arms with respect to baseline characteristics.

**Table 1:** Baseline characteristics of the study population and group comparisons

	No feedback (*n* = 329)	GP-targeted feedback (*n* = 329)		GP-targeted plus patient-targeted feedback (*n* = 329)	
Characteristics	Value	Value	*P*-value^d^	Value	*P*-value^d^
Age, years, mean (s.d.)	39.5 (15.2)	40.1 (15.8)	0.65^a^	39.0 (14.8)	0.62^a^
Gender, %					
Female	64.1	62.3		60.2	
Male	35.6	37.7	0.59^c^	38.9	0.34^c^
Diverse	0.3	0.0	0.86^c^	0.9	0.32^c^
Living situation, %					
With someone	70.6	70.0	0.87^b^	70.4	0.96^b^
Health insurance, %					
Statutory	96.0	95.7	0.85^b^	94.6	0.44^b^
City size, %					
<5000	7.3	6.4		7.1	
5000–19 999	7.6	9.7	0.34^c^	10.2	0.40^c^
20 000–99 999	13.7	10.7	0.76^c^	12.5	0.88^c^
≥100 000	71.4	73.2	0.61^c^	70.3	0.94^c^
School education, %					
≥10 years	83.6	83.3	0.92^b^	84.2	0.83^b^
Baseline in-patient days, mean (s.d.)^e^	2.5 (8.9)	3.1 (12.5)	0.38^a^	1.7 (7.3)	0.34^a^
Baseline number of medications, mean (s.d.)^e^	2.0 (2.4)	2.3 (3.6)	0.29^a^	2.1 (2.5)	0.77^a^
Baseline GP visits, mean (s.d.)^e^	3.8 (4.5)	3.9 (4.5)	0.73^a^	3.7 (5.1)	0.81^a^
EQ-5D index (−0.661 to 1), mean (s.d.)	0.66 (0.28)	0.66 (0.27)	0.98^a^	0.71 (0.24)	0.02^*^^a^
EQ VAS (0–1), mean (s.d.)	0.49 (0.22)	0.51 (0.21)	0.22^a^	0.50 (0.21)	0.45^a^
PHQ-9 at baseline, mean (s.d.)	13.6 (3.4)	13.4 (3.1)	0.47^a^	13.5 (3.3)	0.96^a^

GP, general practitioner; EQ-5D, EuroQol-5D; EQ VAS, EuroQol Visual Analogue Scale; PHQ-9, nine-item Patient Health Questionnaire.

a. Linear ordinary least squares regression.

b. Binary logistic regression.

c. Multinomial logistic regression.

d. *P*-values for GP-targeted feedback and GP-targeted plus patient-targeted feedback are displayed against no feedback as reference group.

e. In the past 6 months.

* *P* < 0.05.

### Base case results

During follow-up, HRQL, as measured by the EQ-5D-5L, increased in all study arms. Depression severity, as assessed by the PHQ-9, decreased in all study groups (Supplementary Material 6). The mean resource use per study arm is available in Supplementary Material 7. Unadjusted cost differences were not statistically significant. The GP-targeted feedback group experienced 0.001 [95% CI: −0.030; +0.029] QALYs less than the no-feedback group, whereas the GP-targeted plus patient-targeted feedback group experienced 0.030 [+0.000; +0.059] QALYs more than the no-feedback group. Unadjusted ICERs revealed that the no-feedback and GP-targeted plus patient-targeted feedback groups dominated the GP-targeted feedback group. GP-targeted plus patient-targeted feedback had slightly higher costs at higher effects than no feedback, yielding an unadjusted ICER of €9 358/QALY.

Regression results for cost categories and effects during follow-up are shown in Table 2. Adjusted cost differences between groups during the 1-year follow-up were not statistically significant, except for psychotherapeutic services. These were taken up more by participants in the GP-targeted feedback group compared with the no-feedback, although the difference was relatively small (fewer than two contacts). Total follow-up costs were highest in the GP-targeted feedback study arm and lowest in the no-feedback arm. Most QALYs occurred in the GP-targeted plus patient-targeted feedback arm, and the fewest in the group with GP-targeted feedback alone. Effect differences were not statistically significant.

**Table 2:** Differences in adjusted costs, QALYs and DFDs between study arms

Category	GP-targeted feedback compared with no feedback; adjusted 12-month differences [95% CI]	GP-targeted plus patient-targeted feedback compared with no feedback; adjusted 12-month differences [95% CI]
Direct costs, €^a^	+560 [−224; +1555]	+666 [−177; +1749]
In-patient services, €^b^	+469 [−1275; +3007]	+465 [−1266; +2976]
Out-patient physician services (without psychotherapy), €^b^	−17 [−162; +150]	+51 [−112; +239]
Out-patient psychiatric and psychotherapy services, €^b^	+451 [+39; +1010]	+362 [−32; +900]
Out-patient non-physician services, €^b^	+53 [−47; +183]	+47 [−58; +185]
Formal nursing care, €^b^	+133 [−101; +1049]	+57 [−109; +597]
Informal care, €^b^	+174 [−603; +1429]	+576 [−388; +2170]
Medication, €^b^	+22 [−13; +73]	+68 [+14; +150]
Indirect costs, €^b^	+98 [−824; +1272]	−247 [−1101; +842]
Societal perspective (base case)		
Total costs to society, €^a^	+542 [−594; +1925]	+376 [−746; +1745]
QALYs^c^	−0.001 [−0.019; +0.017]	+0.003 [−0.016; +0.021]
Payer perspective		
Total payer costs, €^a^	+774 [−1539; +3087]	+868 [−1376: +3113]
QALYs^c^	−0.001 [−0.019; +0.017]	+0.004 [−0.015; +0.023]
Complete case analysis (societal perspective) No feedback: 167; GP-targeted feedback: 158; GP-targeted plus patient-targeted feedback: 176		
Total costs to society, €^a^	−111 [−773; +748]	+218 [−502; +1146]
QALYs^c^	+0.005 [−0.019; +0.028]	+0.008 [−0.015; +0.031]
DFDs as effect measure		
Total costs to society, €^a^	+542 [−594; +1925]	+376 [−746; +1745]
DFDs^c^	−5.4 [−19.2; +8.5]	−3.7 [−17.7; +10.3]

QALYs, quality-adjusted life years; DFDs, depression-free days; GP, general practitioner.

a. Differences in direct costs, societal costs and payer costs were estimated using generalised linear mixed effects models assuming a gamma distribution and a log-link function. Fixed effects were age, gender, education, city size, living situation, baseline health-related quality of life (HRQL), baseline nine-item Patient Health Questionnaire (PHQ-9) score, depression history, pregnancy, breast feeding and baseline healthcare service use (in-patient days, GP visits, number of medications, sick days (not for payer perspective)). Random effects were general practices nested in study centres.

b. Differences in single cost categories as well as indirect costs were estimated using generalised linear mixed effects models assuming a zero-inflated gamma distribution and a log-link function. Fixed effects were age, gender, education, city size, living situation, baseline HRQL, baseline PHQ-9 score, depression history, pregnancy (not for estimation of costs for care), breastfeeding (not for estimation of costs for care) and applicable baseline healthcare service use (in-patient days, GP visits, number of medications, sick days). Random effects were general practices nested in study centres.

c. QALYs and DFDs were estimated via mixed-effects ordinary least squares regressions applying the same covariates as for the estimation of cost differences.

**Fig. 1:**
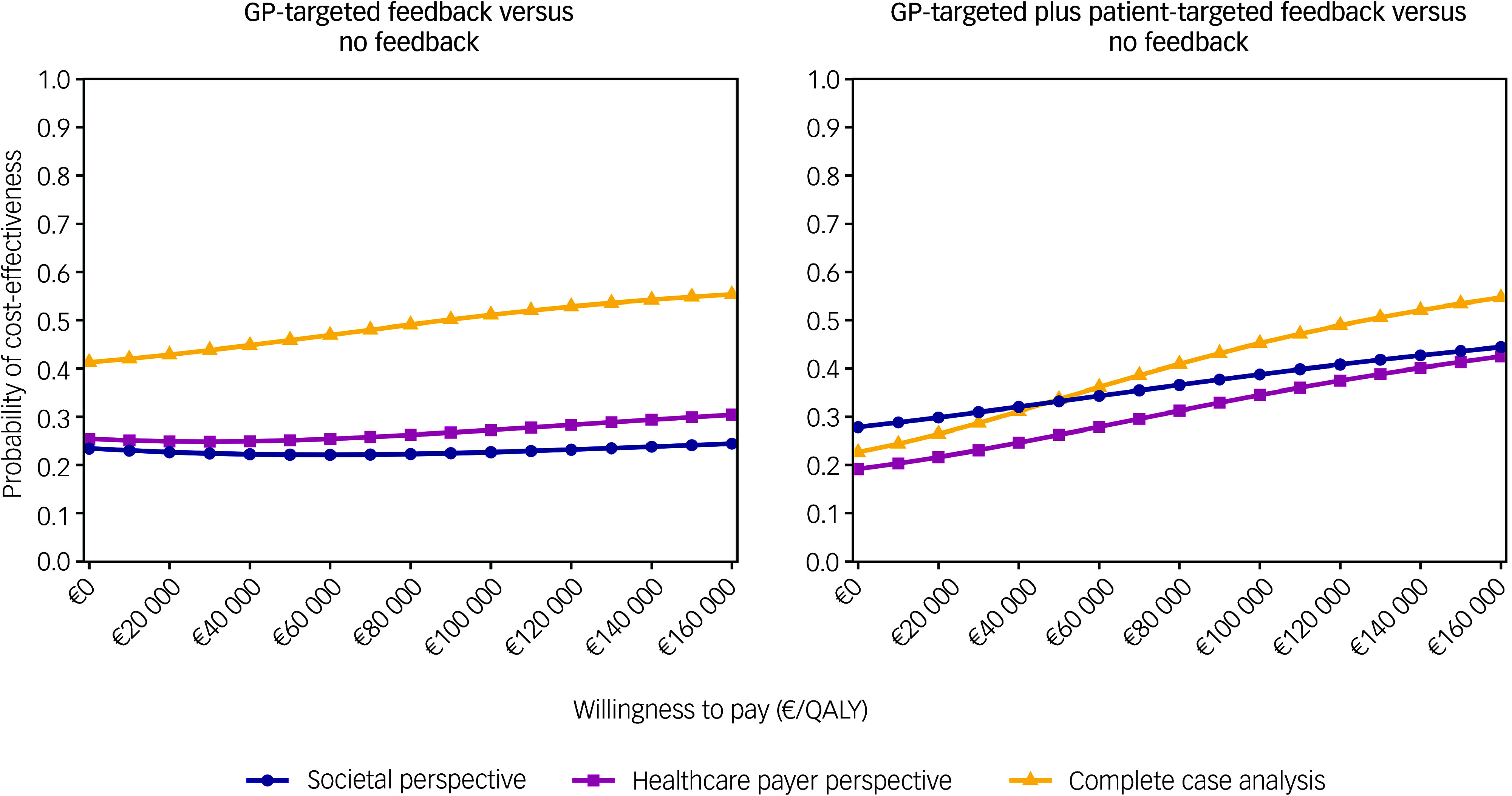
Cost-effectiveness acceptability curves for the base case analysis (societal perspective) and two determinstic sensitivity analyses (healthcare payer perspective and complete case analysis). GP, general practitioner; QALYs, quality-adjusted life years.

The CEACs for the base case analysis showed that GP-targeted feedback achieved low cost-effectiveness probabilities compared with no feedback (22–24%, Fig. 1). The probability of cost-effectiveness of GP-targeted plus patient-targeted feedback ranged between 28 and 44% compared with no feedback.

### Deterministic sensitivity analyses

**Fig. 2:**
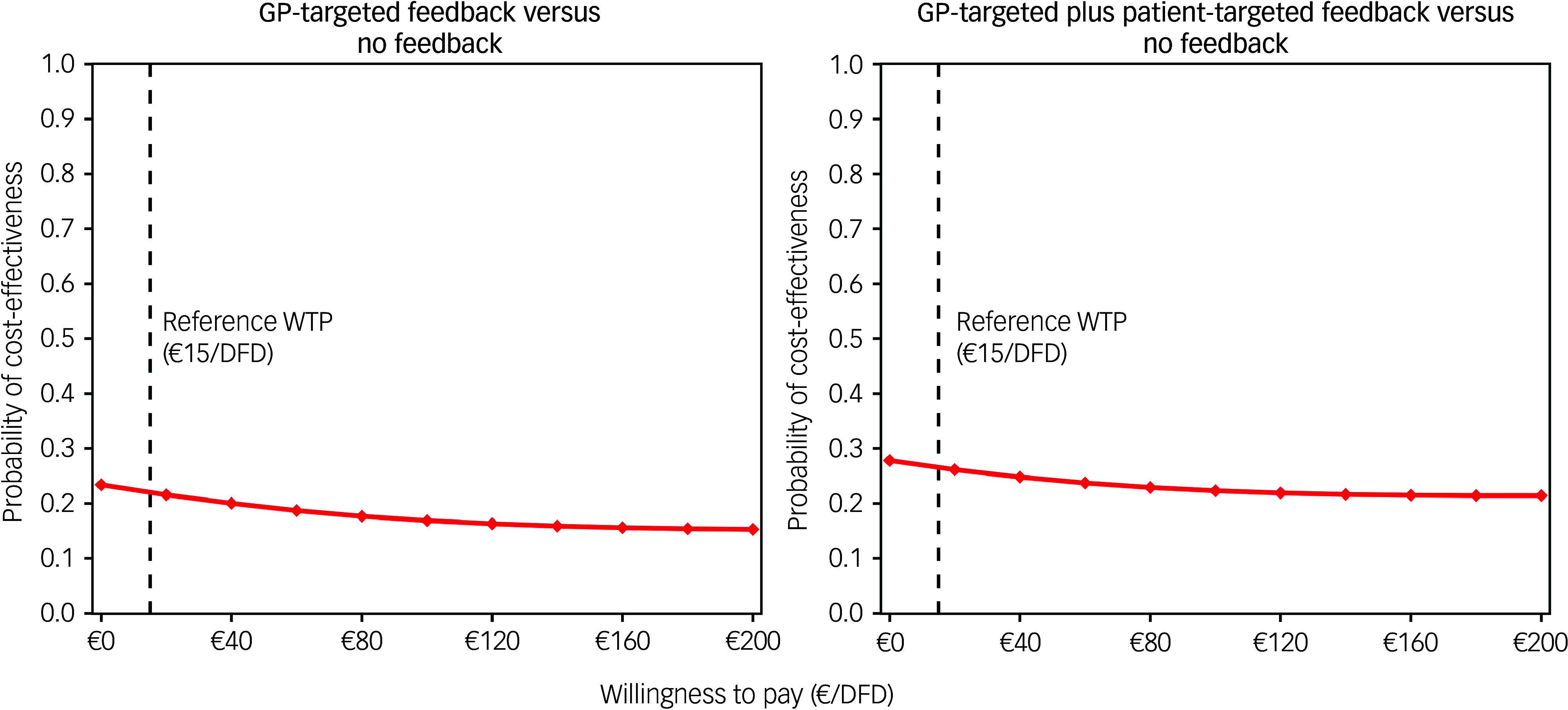
Cost-effectiveness acceptability curves from a societal perspective considering DFDs as effect measure. GP, general practitioner; DFD, depression-free days; WTP, willingness to pay.

From a payer perspective, cost-effectiveness probability trends were similar to the base case; however, they were slightly lower for the GP-targeted plus patient-targeted feedback group (Fig. 1). In the complete case analysis, 501 of 987 participants (51%; no feedback: 167; GP-targeted feedback: 158; GP-targeted plus patient-targeted feedback: 176) were included. In this scenario, adjusted costs and effect differences between study arms were not statistically significant. However, cost-effectiveness probabilities of GP-targeted feedback were higher in this scenario, ranging between 41 and 55%. Cost-effectiveness probabilities of the combined feedback intervention were similar to those obtained in the analysis using multiple imputed data-sets (between 23 and 55%). When DFDs were used as an effect measure, adjusted effects were highest for no feedback and lowest for GP-targeted feedback, although the differences were not statistically significant. CEACs showed low cost-effectiveness probabilities for both interventions compared with no feedback (Fig. 2).

### *Post hoc* explorative subpopulation analyses

Adjusted effect differences of interventions compared with no feedback were not statistically significant (applying a 95% confidence interval) across all subpopulations, or all scenarios for sensitivity analyses. Statistically significant cost differences were observed only in the complete case sample (Supplementary Material 8).

**Fig. 3:**
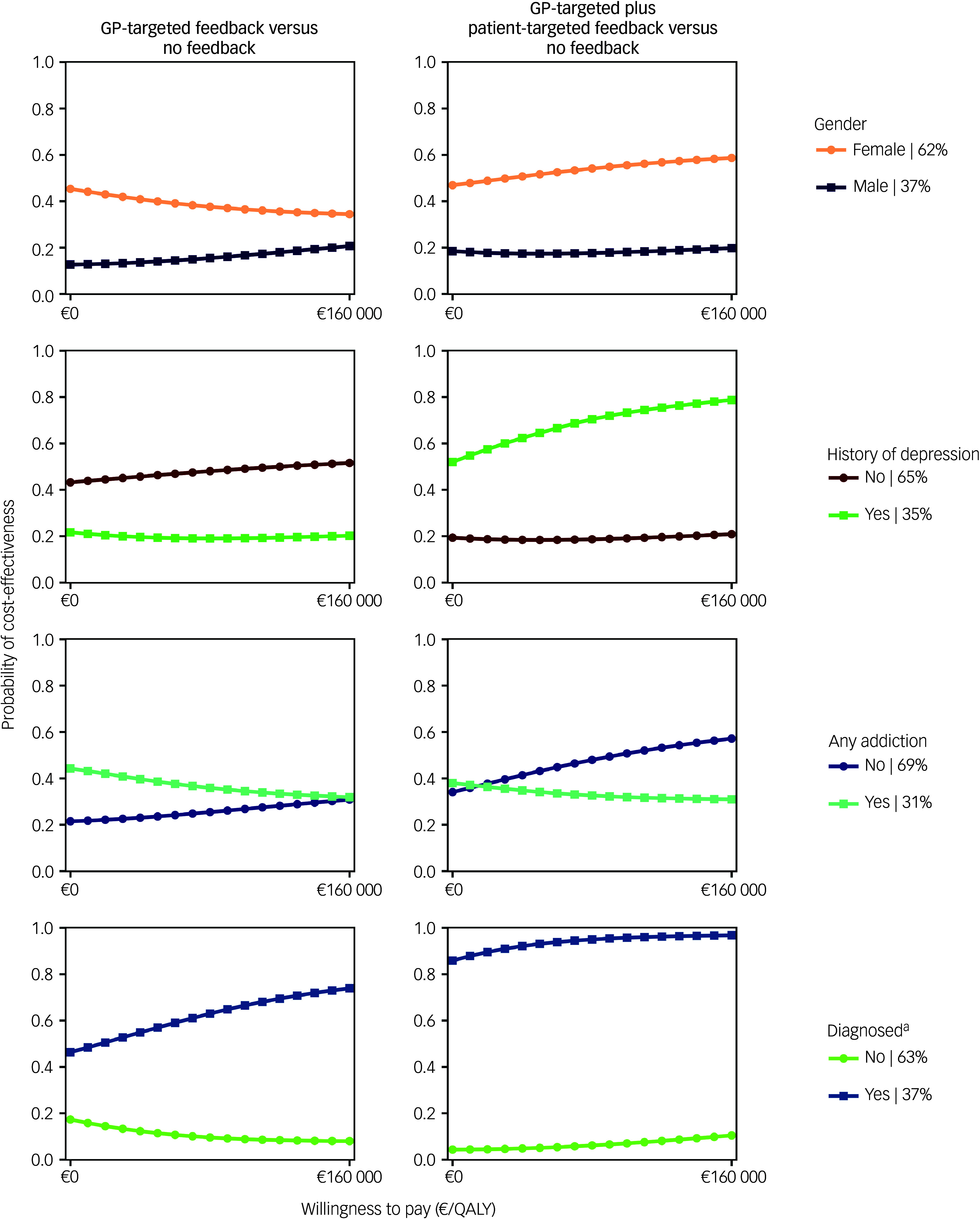
Cost-effectiveness acceptability curves from a societal perspective for selected explorative subpopulation analyses. GP, general practitioner: QALY, quality-adjusted life year. a. The diagnosis criterion was assessed 1 month after baseline using the Mini-International Neuropsychiatric Interview.

CEACs showed that the likelihood of feedback interventions being cost-effective was higher for women and those whose depression diagnosis had been confirmed 1 month after baseline using the MINI (Fig. 3). The combined feedback intervention also had higher cost-effectiveness probabilities for people without an addiction and those with a history of depression.

Cost-effectiveness probabilities for GP-targeted feedback and GP-targeted plus patient-targeted feedback compared with no feedback are presented in Fig. 3. GP-targeted plus patient-targeted feedback yielded the highest cost-effectiveness probabilities in participants with a confirmed depression diagnosis 1 month post-screening (86–97%).

## Discussion

To our knowledge, this work provides the first cost-effectiveness analysis of depression screening feedback interventions targeted at both GPs and patients in a primary care setting. The results of this work showed that the provision of depression screening feedback to patients and/or treating GPs was not significantly associated with any change in overall costs or HRQL. Therefore, we can assume that neither feedback intervention, as it was provided in primary care, was cost-effective from a societal and a healthcare payer perspective. Sample size planning for the GET.FEEDBACK.GP trial was based on depression severity as measured by the PHQ-9.^19^ Feedback interventions showed no statistically significant effect on the PHQ-9.^20^ Although the sample size in this health economic evaluation (*n* = 329 per group) exceeded the initially required sample size for the primary outcome (*n* = 233 per group), it may still have been insufficient to detect differences in costs characterised by high variability and a skewed distribution.^19,47^ Ineffectiveness of the interventions regarding the PHQ-9 could also suggest that no differences in QALYs would be observed even with a larger sample.

A notable finding of our study was the trend towards higher use of out-patient psychotherapy and psychiatric services during follow-up in both the GP-targeted feedback and the combined feedback groups, compared with no feedback. This trend was statistically significant at a 5% significance level for GP-targeted feedback only. For GP-targeted plus patient-targeted feedback, the trend pointed in the same direction but was not statistically significant. This suggests that the provision of feedback after depression screening might be associated with initiation of depression-specific out-patient care. However, this treatment initiation did not translate into significantly better HRQL 12 months after screening. The individual efficacy of depression treatment can sometimes be ascertained years after treatment initiation, as depression frequently manifests as recurrent episodes.^48^ Consequently, the initiation of specialised treatment can be regarded as a strategic investment in one’s future well-being, which can yield favourable health effects in the long run. This, in combination with long waiting times and low rates of patients starting psychotherapy after a first consultation,^49^ advocates for a longer follow-up period to study possible long-term effects of feedback interventions after depression screening.

Another important aspect of the GET.FEEDBACK.GP trial was its setting in primary care. As people access primary care in Germany for various reasons, it was difficult to closely define the sample included in this study. Therefore, the screening in this trial can be considered to have been random (or non-risk-based) screening for a highly diverse sample. As random screening is not associated with improvements in depression outcomes in primary care,^10,11^ it might be difficult to observe positive effects of random screening by adding patient-targeted feedback. These findings were supported by the present study, as well as by the clinical effectiveness results.^20^ The high variability in patient characteristics might have contributed to non-significant group differences. These findings argue in favour of risk-based depression screening including patient-targeted feedback, which seems to be cost-effective for a sample of cardiology patients.^16^

Therefore, we also conducted explorative subpopulation analyses, which led to an interesting finding: high cost-effectiveness probabilities of GP-targeted plus patient-targeted feedback were observed for participants with confirmed depression 1 month after screening, implying that the combined feedback intervention might be cost-effective for individuals whose depressive symptoms are recurring and/or still present 1 month after screening. This highlights the importance of confirming a suspected depressive episode with reliable and well-validated diagnostic interviews to confirm or rule out suspected depression. In addition, it demonstrates a research gap regarding the period between depression screening and official diagnosis, which needs to be investigated further. This finding was also consistent with the results of other subpopulation analyses: probabilities for cost-effectiveness of GP-targeted plus patient-targeted feedback tended to be higher for people with a history of depression. This was in line with national guidelines recommending that screening should be regularly offered to this subpopulation in primary care, as previous depressive episodes are a risk factor for a subsequent depressive episode.^14^ Furthermore, female gender and not having any addiction were associated with higher cost-effectiveness probabilities. This might imply that men and people with an additional addiction might need more intensive follow-up or additional interventions after depression screening. These findings were in line with the primary outcome results, which suggested an association between these factors (female gender, history of depression, no addiction) and decreased depression severity in subgroup analyses.

An alternative approach involves web-based administration of depression screening and feedback interventions, wherein respondents receive automatically tailored feedback on-screen. This approach showed no effects on depression severity 6 months post-screening; however, its economic impact remains to be estimated.^17^

### Strengths and limitations

The trial sample was highly representative of the population with depressive symptoms in Germany. Data from the German National Cohort show that depressive symptoms are more prevalent in females (19.4%) than in males (12.2%), and that the age at the first depressive episode is 36.4 (s.d. = 13.5) years.^1^ In the GET.FEEDBACK.GP sample, the mean age was 39.5 (s.d. = 15.3) years, and 62.2% of participants were female, indicating highly comparable characteristics. This supports depression screening in primary care as an appropriate sector for capturing various pre-existing risk factors with a risk-based screening approach.

This analysis had similar methodological limitations to the clinical effectiveness analysis of GET.FEEDBACK.GP.^20^ These include the lack of a patient-only feedback group and a control group not receiving screening, and the reliance on self-reports.^20^ However, these methodological decisions were intentional to limit the number of study arms, as a non-screened study arm could not deliver the required information, and patient-only feedback was not feasible owing to ethical considerations. By design, there was a high probability that GPs were unblinded regarding the intervention group of their patients during the appointments, which might have influenced their behaviour regarding further diagnostic instruments and the creation of a treatment plan. However, the interventions were designed to trigger a behavioural change both in GPs and patients to increase engagement in depression-specific care. In addition, the interventions intended that either the patient or the GP would address the topic during the consultation.

We identified further limitations applying to the cost-effectiveness analysis. We assumed linear HRQL trends between follow-up periods, which has been common practice in similar health economic evaluations^16^ but might not be adequate for depressive disorders, which can occur as (recurrent) episodes and are associated with mood swings.^14^ In addition, the trial was partly conducted during the COVID-19 pandemic, which might have affected HRQL as well as healthcare availability.^50^ Still, we assumed that these aspects affected all study arms similarly. Last, the sample size in the GET.FEEDBACK.GP trial was planned regarding the primary outcome, PHQ-9, and not cost-effectiveness, for which it is possible that larger sample sizes would have been necessary.

In addition, in this health economic evaluation, multiple imputation by chained equations was used to tackle the challenges of a high proportion of missing data in the original data-set. This approach allowed us to use the whole data-set (except the excluded participants specified in the Methods section) rather than focusing only on a complete case analysis. We observed no significant differences between the characteristics used in the health economic evaluation compared with the clinical effectiveness study.^20^ At the time of analysis, no reliable approach to test the MAR assumption for missing data was available. Therefore, falsifying the MAR hypothesis for the multiple imputation algorithm was infeasible. However, we observed no signs supporting the missing not at random assumption. Hence, we assumed that the MAR hypothesis might hold in this data-set.

An interesting addition to this work would be interpretation of differences in EQ-5D-5L index scores regarding their clinical meaningfulness. However, at present, no minimal clinically important difference for the EQ-5D-5L and the German index scores for people with depression is available. Research using the EQ-5D-3L and its UK value set has suggested an increase of 0.104 to 0.176 as a meaningful difference for a shift to a better health status.^51^ Hypothetically, we therefore would not observe a meaningful difference at any measurement point between the three study arms. However, no feedback and GP-targeted feedback might on average be associated with a shift to a better health status, as the average EQ-5D-5L index increased by 0.15 (no feedback) and 0.13 (GP-targeted feedback) over the course of the follow-up.

It is important to acknowledge the limitations of the *post hoc* explorative subpopulation analyses performed here. As the GET.FEEDBACK.GP trial was only stratified by the treating GP and depression severity as assessed by the PHQ-9,^20^ the trial was not powered for further subpopulation analyses. Consequently, the sample was not balanced regarding the categories for which we conducted subpopulation analyses (gender, depression history, addiction, confirmed depression using the MINI). Hence, one must be careful when drawing conclusions from these results, as they might not be suitable for definite decision-making. However, they could inform the scientific community about potential target populations and suggest further research directions regarding what feedback to provide to whom after depression screening. They also highlight the importance of following up on a depression screening with a diagnostic interview (e.g. the MINI).

The estimation of costs from healthcare service use was based on standardised unit costs from a societal perspective, which are defined as mean costs per unit. Therefore, differences in the duration of GP contacts as a consequence of feedback provided to one or more involved parties could not be captured in our analysis, increasing the costs of feedback interventions and leading to possibly lower cost-effectiveness probabilities.

Finally, future research should also investigate the activation of specialised psychological treatment after depression screening and its possible lagged effects on depression outcomes.

## Supporting information

Kreis et al. supplementary material 1 Kreis et al. supplementary material

Kreis et al. supplementary material 2 Kreis et al. supplementary material

Kreis et al. supplementary material 3 Kreis et al. supplementary material

Kreis et al. supplementary material 4 Kreis et al. supplementary material

Kreis et al. supplementary material 5 Kreis et al. supplementary material

Kreis et al. supplementary material 6 Kreis et al. supplementary material

Kreis et al. supplementary material 7 Kreis et al. supplementary material

Kreis et al. supplementary material 8 Kreis et al. supplementary material

## Data Availability

The data supporting the findings of this study are available on request from the corresponding author, L.G.K.

## Supplementary material

The supplementary material is available online at https://doi.org/10.1192/bjo.2025.10945
