# Supplementary figures and images for: Cost-effectiveness of targeted feedback interventions after depression screening in primary care: health economic evaluation of the GET.FEEDBACK.GP trial
Source: BJPsych Open. 2026 Feb 2;12(2):e52. doi: 10.1192/bjo.2025.10945 (PMC12926889; doi:10.1192/bjo.2025.10945)

**Supplementary Material 2:**

Participants flowchart


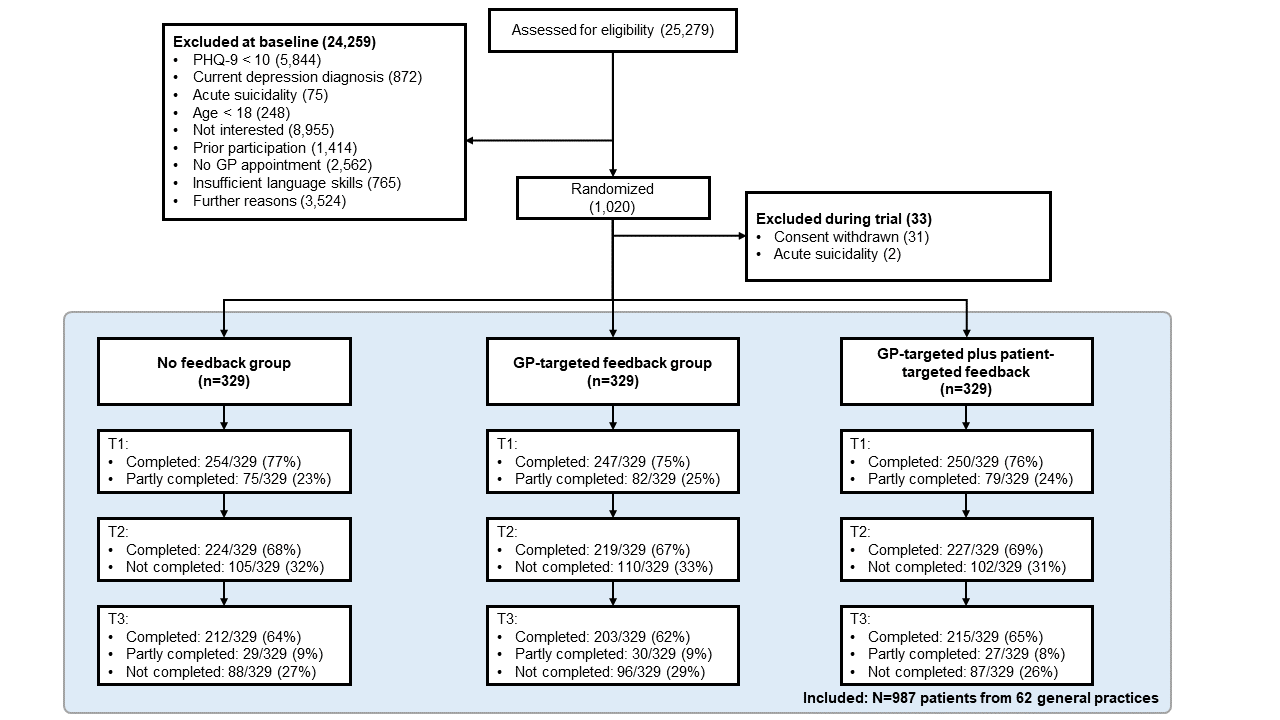

Supplement: Kreis et al. supplementary material 2 — Kreis et al. supplementary material [file S2056472425109459sup002.docx]
