# Supplementary material for: Cost-effectiveness of targeted feedback interventions after depression screening in primary care: health economic evaluation of the GET.FEEDBACK.GP trial
Source: BJPsych Open. 2026 Feb 2;12(2):e52. doi: 10.1192/bjo.2025.10945 (PMC12926889; doi:10.1192/bjo.2025.10945)
Supplement: Kreis et al. supplementary material 3 — Kreis et al. supplementary material [file S2056472425109459sup003.docx]

**Supplementary Material 3: Intervention costs**

In this analysis, intervention costs were disregarded as differences between study arms were negligibly small. However, if depression screening followed by a feedback intervention were to be implemented in primary care, additional costs would arise.

Fixed costs would arise to acquire 2 tablets per general practice. These costs can be estimated to 860€ (850€ for two tablets, 10€ for electricity) per general practice [e]. Fixed costs for screening per patient (irrespective of the feedback intervention) can be assumed to 0.52€, as one general practice covers care for approx. 1,657 adults (55.1 million adults in Germany with at least 1 GP contact per year divided by 33,218 primary care practices in Germany) [a,b,c].

Variable costs are the costs of printing (printer use and paper) to provide feedback to GPs or patients, whereas in no feedback, no printing costs would arise, in GP-targeted feedback 1 sheet of paper is printed, and in GP-targeted plus patient-targeted feedback 2 sheets are printed. Assuming quarterly screening (4 GP contacts per year [d]), yearly printing costs per patient would be 0.00€ for no feedback, 0.40€ for GP-targeted feedback, and 0.80€ for GP-targeted plus patient targeted feedback. Additional personnel costs for the administration of screening and feedback interventions would arise. We assumed approx. 3 minutes per patient for handing out and receiving back the tablet, providing technical assistance, and operating the printer. These tasks would be covered by physician assistants (*Medizinische Fachangestellte*) in the practice. We assumed a median wage for physician assistants of 3,129€ per month [f] and a weekly worktime of 38.5 hours (monthly: 38.5 hours x 4.33 weeks = 166.7 hours). Hence, 3 minutes would be priced at approx. 0.94 €, resulting in yearly costs per patient of 3.75€ (in total 12 minutes in 4 GP contacts per year).

Summing up fixed and variable costs, total intervention costs per patient per year would be 4.27€ for no feedback, 4.67€ for GP-targeted feedback, and 5.07€ for GP-targeted plus patient-targeted feedback.

Yearly costs for outpatient physician care in Germany are approx. 360€ per person [g]. Thereof, intervention costs would be 1.19% for no feedback, 1.30% for GP-targeted feedback only, and 1.41% for GP-targeted plus patient-targeted feedback.

|  | **Item** | **Quantity** | **Item costs** | **Yearly costs per general practice** | **Yearly costs per patient** | **Source(s)** |
| --- | --- | --- | --- | --- | --- | --- |
| Number of patients | Adults in Germany | 69,602,098 |  |  |  | a |
|  | Adults in Germany with at least one GP visit per year | 55,055,260 (79.1% of adults) |  |  |  | a, b |
|  | General practices in Germany | 33,218 |  |  |  | c |
|  | Number of patients per general practice | 1,657 |  |  |  | a, b, c |
|  | Median GP visits per patient per year | 4 |  |  |  | d |
| Costs for technology and personnel | Tablet | 2 | 425 € | 850 € | 0.51 € | e |
|  | Yearly charging costs | 2 | 5 € | 10 € | 0.01 € | Assumption |
|  | Personnel costs for administration | 12 min | 0.31 € | 6,213.75 € | 3.75 € | f |
|  | Printing costs (no feedback) | 0 | 0.10 € | 0 € | 0 € | Assumption |
|  | Printing costs (GP-feedback) | 1 | 0.10 € | 662.80 € | 0.40 € | Assumption |
|  | Printing costs (GP-patient feedback) | 2 | 0.10 € | 1,325.60 € | 0.80 € | Assumption |
| **Total costs** | **Total costs (no feedback)** |  |  | 7,073.75 € | 4.27 € | Calculation |
|  | **Total costs (GP-feedback)** |  |  | 7,736.55 € | 4.67 € | Calculation |
|  | **Total costs (GP-patient feedback)** |  |  | 8,399.35 € | 5.07 € | Calculation |

**Sources:**

1. <https://gesundheitsdaten.kbv.de/cms/html/17020.php>; Access date: 24.09.2025
2. Robert Koch-Institut. Inanspruchnahme allgemeinärztlicher Leistungen (ab 18 Jahre). Gesundheitsberichterstattung des Bundes. 2024 [Access date: 24.09.2025]; Available from: <https://gbe.rki.de>
3. <https://gesundheitsdaten.kbv.de/cms/html/17020.php>; Access date: 24.09.2025
4. <https://gesundheitsdaten.kbv.de/cms/html/24044.php>; Access date: 24.09.2025
5. <https://de.statista.com/statistik/daten/studie/290238/umfrage/durchschnittspreise-fuer-tablets-in-deutschland/>; Access date: 24.09.2025
6. <https://web.arbeitsagentur.de/entgeltatlas/beruf/33213>; Access date: 02.12.2025
7. <https://www.destatis.de/DE/Themen/Gesellschaft-Umwelt/Gesundheit/Krankheitskosten/Tabellen/einrichtungen-geschlecht.html>; Access date: 24.09.2025
