# Supplementary material for: Cost-effectiveness of targeted feedback interventions after depression screening in primary care: health economic evaluation of the GET.FEEDBACK.GP trial
Source: BJPsych Open. 2026 Feb 2;12(2):e52. doi: 10.1192/bjo.2025.10945 (PMC12926889; doi:10.1192/bjo.2025.10945)
Supplement: Kreis et al. supplementary material 4 — Kreis et al. supplementary material [file S2056472425109459sup004.docx]

**Supplementary Material 4:**

Unit costs

| **Cost category** | **Cost item** | **Price (2022-€)** | **Source** |
| --- | --- | --- | --- |
| Inpatient stays (per day) | Inpatient stay general hospital (per day) | 1114,41 | a |
|  | Day-care general hospital (per day) | 724,37 | a |
|  | Inpatient stay psychiatric hospital (per day) | 473,47 | a |
|  | Day-care psychiatric hospital (per day) | 360,16 | a |
|  | Inpatient stay rehabilitation clinic (per day) | 203,57 | a |
|  | Day-care rehabilitation clinic (per day) | 82,56 | a |
| Outpatient physician visits (per contact) | General practitioner | 28,30 | a |
|  | Internist | 80,55 | a |
|  | Cardiologist | 80,55 | a |
|  | Pulmonologist | 80,55 | a |
|  | Endocrinologist | 80,55 | a |
|  | Diabetologist | 80,55 | a |
|  | Nephrologist | 80,55 | a |
|  | Orthopedist | 35,02 | a |
|  | Psychiatrist | 66,78 | b |
|  | Neurologist | 66,82 | a |
|  | Dermatologist | 29,96 | a |
|  | Ear-nose-throat (ENT) specialist | 44,71 | a |
|  | Outpatient surgeon | 61,53 | a |
|  | Radiologist | 66,78 | b |
|  | Urologist | 32,76 | a |
|  | Ophthalmologist | 57,20 | a |
|  | Gynecologist | 45,56 | a |
|  | Rheumatologist | 80,55 | a |
|  | Dentist / Orthodentist | 58,21 | a |
|  | Emergency physician | 658,34 | c |
|  | Hospital outpatient clinic | 724,37 | a |
|  | Emergency service (116 117) | 201,26 | d |
|  | Further specialist | 55,12 | b |
|  | Psychologist / Psychotherapist | 113,37 | a |
| Non-medical services (per contact) | Ergotherapy | 59,71 | a |
|  | Alternative practitioner | 29,43 | a |
|  | Inhalation therapy | 29,43 | a |
|  | Physiotherapy | 23,63 | a |
|  | Massage or lymphatic drainage | 29,43 | a |
|  | Sports or exercise therapy | 23,63 | a |
|  | Speech therapy | 66,86 | a |
|  | Heat, cold and water therapy | 29,43 | a |
|  | Self-help groups | 29,43 | a |
|  | Further non-medical services | 29,43 | a |
| Care | Professional care (per minute) | 0,59 | b |
|  | Informal care (per h, replacement cost approach, gross wage labour costs for the economic sector social work (excl. homes) plus non-wage labour costs) | 29,14 | b,e,f |
|  | Contact lump sum (per contact) | 3,88 | b |
| Productivity losses | Sick leave | 208,66 | g |
|  | Hours spent for physician visits | 29,55 | h |

References:

1. Muntendorf L-K, Brettschneider C, Konnopka A, König H-H. [Updating standardized unit costs from a societal perspective for health economic evaluation] Aktualisierung der standardisierten Bewertungssätze aus gesellschaftlicher Perspektive für gesundheitsökonomische Evaluationen. *Gesundheitswesen* 2024; 86(05): 389-393.
2. Bock JO, Brettschneider C, Seidl H*, et al.* Calculation of Standardised Unit Costs from a Societal Perspective for Health Economic Evaluation. *Gesundheitswesen* 2015; 77(01): 53-61.
3. <https://www.vdek.com/LVen/HAM/fokus/Rettungsdienst/RettungsdienstKosten.html#:~:text=Der%20Rettungsdienst%20ist%20im%20neuen,von%20534%20auf%20616%20Euro>. (accessed on May 19, 2025)
4. Mayer S, Berger M, Konnopka A*, et al.* In Search for Comparability: The PECUNIA Reference Unit Costs for Health and Social Care Services in Europe. International Journal of Environmental Research and Public Health, 2022. 19, DOI: 10.3390/ijerph19063500.
5. <https://www.statistischebibliothek.de/mir/receive/DEHeft_mods_00141316> (accessed on May 26, 2025)
6. <https://www.destatis.de/DE/Themen/Arbeit/Arbeitskosten-Lohnnebenkosten/Tabellen/lohnkosten-deutschland.html> (accessed on May 26, 2025)
7. <https://www.destatis.de/DE/Themen/Arbeit/Verdienste/Verdienste-Branche-Berufe/_inhalt.html> (accessed May 19, 2025)
8. <https://www.destatis.de/Europa/DE/Thema/Bevoelkerung-Arbeit-Soziales/Arbeitsmarkt/Wochenarbeitszeiten.html#:~:text=2021%20arbeiteten%20in%20der%20EU,40%2C6%20Stunden%20leicht%20dar%C3%BCber>. (accessed May 19, 2025)
