# Supplementary material for: Cost-effectiveness of targeted feedback interventions after depression screening in primary care: health economic evaluation of the GET.FEEDBACK.GP trial
Source: BJPsych Open. 2026 Feb 2;12(2):e52. doi: 10.1192/bjo.2025.10945 (PMC12926889; doi:10.1192/bjo.2025.10945)

**Supplementary Material 5: Report on missing data**

1. Share of missing data by baseline input variable

| **Input variable** | **Category** | **Share of NA in outcome variables** | **Input variable** | **Category** | **Share of NA in outcome variables** |
| --- | --- | --- | --- | --- | --- |
| General Practice ID | 1 | 29,5% | t0_EQ5D_1  (Mobility) | 0 | 24,2% |
|  | 2 | 26,0% |  | 1 | 29,2% |
|  | 3 | 36,7% |  | 2 | 26,8% |
|  | 4 | 46,3% |  | 3 | 32,7% |
|  | 5 | 25,9% |  | 4 | 54,6% |
|  | 6 | 35,3% | t0_EQ5D_2 (Self-care) | 0 | 25,3% |
|  | 8 | 55,3% |  | 1 | 26,5% |
|  | 9 | 41,0% |  | 2 | 44,2% |
|  | 10 | 19,8% |  | 3 | 41,9% |
|  | 11 | 29,3% |  | 4 | 51,3% |
|  | 12 | 31,9% | t0_EQ5D_3 (Usual activities) | 0 | 24,5% |
|  | 13 | 16,1% |  | 1 | 27,4% |
|  | 14 | 47,8% |  | 2 | 27,1% |
|  | 15 | 53,4% |  | 3 | 30,0% |
|  | 16 | 22,7% |  | 4 | 25,8% |
|  | 17 | 32,1% | t0_EQ5D_4 (Pain/discomfort) | 0 | 24,2% |
|  | 18 | 67,0% |  | 1 | 28,1% |
|  | 19 | 23,1% |  | 2 | 24,6% |
|  | 20 | 39,5% |  | 3 | 28,8% |
|  | 21 | 21,7% |  | 4 | 33,6% |
|  | 22 | 9,6% | t0_EQ5D_5 (Anxiety/depression) | 0 | 29,9% |
|  | 23 | 18,3% |  | 1 | 25,2% |
|  | 25 | 27,1% |  | 2 | 25,0% |
|  | 26 | 22,0% |  | 3 | 27,0% |
|  | 27 | 19,6% |  | 4 | 37,5% |
|  | 28 | 51,7% | Sick leave period in the last 6 months | 0 days | 27,0% |
|  | 29 | 95,6% |  | 1 week | 27,7% |
|  | 30 | 36,0% |  | 2-3 weeks | 34,2% |
|  | 31 | 17,5% |  | 1-2 month(s) | 35,6% |
|  | 32 | 36,1% |  | 3-4 months | 49,9% |
|  | 33 | 28,6% |  | > 4 months | 35,1% |
|  | 34 | 41,2% | Risk factor (RF): Reason for GP visit | Physical symptoms | 30,2% |
|  | 35 | 6,2% |  | Mental symptoms | 31,6% |
|  | 36 | 32,4% |  | Neither | 29,6% |
|  | 37 | 5,3% | RF: Anxieties | No | 25,6% |
|  | 38 | 36,3% |  | Yes | 26,7% |
|  | 39 | 8,7% | RF: Addiction | No | 23,9% |
|  | 40 | 22,0% |  | Yes | 30,8% |
|  | 41 | 8,4% | RF: Traumatic event | No | 25,4% |
|  | 42 | 22,0% |  | Yes | 26,6% |
|  | 43 | 15,1% | RF: Persistent somatic symptoms | No | 23,5% |
|  | 44 | 57,1% |  | Yes | 28,1% |
|  | 45 | 30,7% | RF: Mood swings | No | 34,9% |
|  | 46 | 13,6% |  | Yes | 25,3% |
|  | 47 | 57,8% | RF: Chronic physical disease | No | 26,9% |
|  | 48 | 26,5% |  | Yes | 24,9% |
|  | 49 | 21,0% | RF: Social support | No | 34,5% |
|  | 50 | 14,3% |  | Yes | 23,5% |
|  | 51 | 17,6% | Previous depression diagnosis | Never | 27,5% |
|  | 52 | 28,2% |  | 1-5 years ago | 21,9% |
|  | 53 | 11,2% |  | 5-10 years ago | 27,8% |
|  | 54 | 38,0% |  | > 10 years ago | 20,4% |
|  | 55 | 18,2% | Previous depression treatment | Never | 28,1% |
|  | 56 | 39,7% |  | 1-5 years ago | 20,8% |
|  | 57 | 28,3% |  | 5-10 years ago | 30,7% |
|  | 58 | 64,6% |  | > 10 years ago | 19,6% |
|  | 59 | 34,1% | Treatment type | *No treatment* | 31,0% |
|  | 60 | 35,9% |  | Psychotherapy | 39,2% |
|  | 61 | 14,6% |  | Antidepressants | 55,8% |
|  | 62 | 11,4% |  | PT + AD | 36,2% |
|  | 63 | 32,4% | Diagnosis: Who? | *No diagnosis* | 28,5% |
|  | 64 | 30,4% |  | GP | 28,8% |
| City size | <5 000 | 27,7% |  | Psychotherapist | 30,2% |
|  | 5 000 - 19 999 | 42,3% |  | Psychiatrist | 33,1% |
|  | 20 000 – 99 999 | 31,9% |  | Neurologist | 43,7% |
|  | ≥ 100 000 | 24,6% |  | Other physician | 44,3% |
| Mother tongue | German | 24,1% | RF: Other mental disorder | Never | 26,8% |
|  | Other | 40,2% |  | In the last year | 20,9% |
| Self-definition as migrant | No | 26,0% |  | 1-5 years ago | 26,9% |
|  | Yes | 26,9% |  | 5-10 years ago | 31,0% |
| Perceived by others as migrant | No | 25,1% |  | > 10 years ago | 26,9% |
|  | Yes | 31,1% | RF: Mental disorder in the family | No | 27,4% |
| Gender | Female | 24,8% |  | Yes | 25,1% |
|  | Male | 28,1% | RF: Suicidal ideation in the family | No | 26,6% |
|  | Diverse | 20,8% |  | Yes | 24,1% |
| Marital status | Unwed | 30,3% | RF: Pregnancy planned | No | 26,6% |
|  | Married | 21,6% |  | Yes | 37,0% |
|  | Divorced | 30,0% | RF: Currently pregnant | No | 26,6% |
|  | Widowed | 35,2% |  | Yes | 67,4% |
|  | In partnership | 27,8% | RF: Gave birth in the last 6 months | No | 26,4% |
|  | Other | 39,3% |  | Yes | 84,5% |
| Living situation | With someone | 25,6% | RF: Currently breastfeeding | No | 26,6% |
|  | Alone | 29,8% |  | Yes | 58,1% |
| Highest schooling degree | In school | 40,8% | RF: PMS | No | 26,5% |
|  | *Sonderschulabschluss* | 49,6% |  | Yes | 29,2% |
|  | *Hauptschulabschluss* | 38,2% | RF: Menopausal | No | 28,0% |
|  | *Mittlere Reife* | 30,1% |  | Yes | 21,6% |
|  | *Fachabitur* | 26,7% | t0_PHQ9_1 | 0 | 37,7% |
|  | *Abitur* | 18,8% |  | 1 | 24,0% |
| Level of education | No education degree | 33,0% |  | 2 | 25,8% |
|  | In education | 35,7% |  | 3 | 28,9% |
|  | *Lehre/Ausbildung/Fachschule* | 29,5% | t0_PHQ9_2 | 0 | 26,4% |
|  | *Meister* | 34,5% |  | 1 | 23,3% |
|  | University | 18,7% |  | 2 | 25,9% |
|  | Other | 39,4% |  | 3 | 34,5% |
| Employment status | Not in employment | 28,0% | t0_PHQ9_3 | 0 | 14,7% |
|  | Retired | 28,0% |  | 1 | 23,8% |
|  | Unemployed | 38,0% |  | 2 | 25,7% |
|  | Part-time employment | 21,5% |  | 3 | 29,1% |
|  | Full-time emplyoment | 26,7% | t0_PHQ9_4 | 0 | 25,0% |
| Smoking | No | 22,6% |  | 1 | 26,3% |
|  | Yes | 32,0% |  | 2 | 26,1% |
| Alcohol drinking frequency | Never | 31,0% |  | 3 | 26,5% |
|  | 1 time / month | 28,3% | t0_PHQ9_5 | 0 | 25,2% |
|  | 1-4 times / month | 22,8% |  | 1 | 25,5% |
|  | 1-3 times / week | 22,5% |  | 2 | 24,6% |
|  | ≥ 4 times / week | 34,0% |  | 3 | 30,4% |
| If alcohol consumption: How much? | *No* *alcohol* | 31,3% | t0_PHQ9_6 | 0 | 29,4% |
|  | 1-2 drinks | 25,0% |  | 1 | 25,1% |
|  | 3-4 drinks | 25,6% |  | 2 | 24,1% |
|  | 5-6 drinks | 30,5% |  | 3 | 27,5% |
|  | 7-8 drinks | 29,0% | t0_PHQ9_7 | 0 | 27,2% |
|  | ≥ 9 drinks | 50,5% |  | 1 | 24,6% |
| Binge alcohol drinking (≥ 6 drinks) | Never | 55,9% |  | 2 | 28,4% |
|  | ≤ 1 time / month | 56,7% |  | 3 | 24,6% |
|  | 1 time / month | 75,6% | t0_PHQ9_8 | 0 | 22,3% |
|  | 1 time / week | 84,5% |  | 1 | 28,0% |
|  | (nearly) daily | 94,5% |  | 2 | 31,6% |
| t0_GAD7_1 | 0 | 32,7% |  | 3 | 35,5% |
|  | 1 | 26,9% | t0_PHQ9_9 | 0 | 25,0% |
|  | 2 | 24,0% |  | 1 | 26,8% |
|  | 3 | 26,7% |  | 2 | 34,8% |
| t0_GAD7_2 | 0 | 30,4% | Study center | HD | 30,6% |
|  | 1 | 24,9% |  | HH | 20,4% |
|  | 2 | 25,7% |  | JE | 33,5% |
|  | 3 | 28,1% |  | MU | 29,9% |
| t0_GAD7_3 | 0 | 28,6% |  | TÜ | 28,1% |
|  | 1 | 25,3% | Feedback group | No feedback | 25,5% |
|  | 2 | 26,5% |  | GP-feedback | 27,2% |
|  | 3 | 28,0% |  | GP-patient-feedback | 25,4% |
| t0_GAD7_4 | 0 | 28,8% |  |  |  |
|  | 1 | 26,1% |  |  |  |
|  | 2 | 25,5% |  |  |  |
|  | 3 | 27,0% |  |  |  |
| t0_GAD7_5 | 0 | 26,1% |  |  |  |
|  | 1 | 24,9% |  |  |  |
|  | 2 | 28,7% |  |  |  |
|  | 3 | 28,4% |  |  |  |
| t0_GAD7_6 | 0 | 26,1% |  |  |  |
|  | 1 | 25,5% |  |  |  |
|  | 2 | 26,5% |  |  |  |
|  | 3 | 28,2% |  |  |  |
| t0_GAD7_7 | 0 | 24,2% |  |  |  |
|  | 1 | 24,5% |  |  |  |
|  | 2 | 27,5% |  |  |  |
|  | 3 | 32,5% |  |  |  |
| t0_SSS8_1 | 0 | 21,5% |  |  |  |
|  | 1 | 26,2% |  |  |  |
|  | 2 | 27,2% |  |  |  |
|  | 3 | 29,4% |  |  |  |
|  | 4 | 29,2% |  |  |  |
| t0_SSS8_2 | 0 | 20,5% |  |  |  |
|  | 1 | 24,6% |  |  |  |
|  | 2 | 25,2% |  |  |  |
|  | 3 | 28,6% |  |  |  |
|  | 4 | 33,1% |  |  |  |
| t0_SSS8_3 | 0 | 25,7% |  |  |  |
|  | 1 | 26,9% |  |  |  |
|  | 2 | 22,0% |  |  |  |
|  | 3 | 30,0% |  |  |  |
|  | 4 | 30,1% |  |  |  |
| t0_SSS8_4 | 0 | 22,9% |  |  |  |
|  | 1 | 23,8% |  |  |  |
|  | 2 | 28,4% |  |  |  |
|  | 3 | 30,1% |  |  |  |
|  | 4 | 31,5% |  |  |  |
| t0_SSS8_5 | 0 | 25,0% |  |  |  |
|  | 1 | 24,5% |  |  |  |
|  | 2 | 27,0% |  |  |  |
|  | 3 | 27,5% |  |  |  |
|  | 4 | 34,3% |  |  |  |
| t0_SSS8_6 | 0 | 22,4% |  |  |  |
|  | 1 | 25,7% |  |  |  |
|  | 2 | 31,4% |  |  |  |
|  | 3 | 31,0% |  |  |  |
|  | 4 | 26,9% |  |  |  |
| t0_SSS8_7 | 0 | 36,1% |  |  |  |
|  | 1 | 36,1% |  |  |  |
|  | 2 | 25,2% |  |  |  |
|  | 3 | 27,2% |  |  |  |
|  | 4 | 23,8% |  |  |  |
| t0_SSS8_8 | 0 | 23,6% |  |  |  |
|  | 1 | 18,3% |  |  |  |
|  | 2 | 25,4% |  |  |  |
|  | 3 | 28,1% |  |  |  |
|  | 4 | 30,9% |  |  |  |

1. Regression results

Univariate binary logistic regressions were specified with the binary missingness indicator of the outcome variable of the health economic evaluation (total societal costs and QALYs) as the dependent variable. The baseline variables from the previous table were sequentially used as predictor variables. Odds Ratios (ORs) above 1 mean higher likelihood of missingness.

| **Predictors** | **Missing societal costs at 1-year FU** | | | **Missing QALYs at 1-year FU** | | |
| --- | --- | --- | --- | --- | --- | --- |
|  | **OR** | **95% CI** | **p-value** | **OR** | **95% CI** | **p-value** |
| **General Practice ID (reference: 1)** | | | | | | |
| 2 | 0.59 | 0.07, 3.73 | 0.571 | 0.76 | 0.10, 4.80 | 0.767 |
| 3 | 2.42 | 0.76, 9.34 | 0.157 | 1.30 | 0.43, 4.03 | 0.645 |
| 4 | 1.98 | 0.59, 7.81 | 0.286 | 1.32 | 0.40, 4.45 | 0.640 |
| 5 | 0.50 | 0.12, 1.80 | 0.303 | 0.43 | 0.09, 1.59 | 0.231 |
| 6 | 1.23 | 0.36, 4.48 | 0.738 | 1.59 | 0.47, 5.77 | 0.460 |
| 8 | 5.29 | 0.85, 102 | 0.131 | 6.81 | 1.09, 132 | 0.082 |
| 9 | 1.76 | 0.51, 7.04 | 0.385 | 1.59 | 0.47, 5.77 | 0.460 |
| 10 | 1.47 | 0.34, 7.55 | 0.615 | 0.68 | 0.13, 2.97 | 0.615 |
| 11 | 0.63 | 0.17, 2.14 | 0.460 | 0.57 | 0.14, 1.96 | 0.385 |
| 12 | 0.44 | 0.02, 4.78 | 0.510 | 0.57 | 0.03, 6.16 | 0.649 |
| 13 | 0.50 | 0.12, 1.80 | 0.303 | 0.43 | 0.09, 1.59 | 0.231 |
| 14 | 0.88 | 0.03, 22.8 | 0.929 | 1.14 | 0.04, 29.4 | 0.929 |
| 15 | 1.76 | 0.16, 38.8 | 0.649 | 2.27 | 0.21, 50.0 | 0.510 |
| 16 | 0.66 | 0.12, 3.18 | 0.603 | 0.45 | 0.06, 2.25 | 0.362 |
| 17 | 0.98 | 0.36, 2.71 | 0.967 | 0.66 | 0.23, 1.82 | 0.433 |
| 18 | 5,072,359 | 0.00, NA | 0.979 | 5.68 | 0.86, 111 | 0.121 |
| 19 | 1.04 | 0.42, 2.64 | 0.931 | 0.68 | 0.26, 1.72 | 0.422 |
| 20 | 7.05 | 1.21, 134 | 0.072 | 3.97 | 0.89, 27.8 | 0.098 |
| 21 | 0.62 | 0.27, 1.38 | 0.244 | 0.47 | 0.19, 1.09 | 0.088 |
| 22 | 0.29 | 0.10, 0.78 | **0.019** | 0.30 | 0.09, 0.83 | **0.028** |
| 23 | 0.59 | 0.14, 2.21 | 0.436 | 0.49 | 0.10, 1.89 | 0.321 |
| 25 | 1.06 | 0.41, 2.78 | 0.909 | 0.95 | 0.36, 2.44 | 0.909 |
| 26 | 1.08 | 0.46, 2.58 | 0.853 | 1.06 | 0.45, 2.49 | 0.894 |
| 27 | 0.51 | 0.17, 1.41 | 0.206 | 0.66 | 0.23, 1.82 | 0.433 |
| 28 | 2.06 | 0.53, 10.1 | 0.321 | 2.65 | 0.68, 13.0 | 0.180 |
| 29 | 5,072,359 | 0.00, NA | 0.992 | 2,404,421 | 0.00, NA | 0.987 |
| 30 | 1.41 | 0.43, 5.02 | 0.576 | 1.82 | 0.56, 6.47 | 0.330 |
| 31 | 0.45 | 0.22, 0.92 | **0.031** | 0.45 | 0.21, 0.93 | **0.034** |
| 32 | 1.36 | 0.57, 3.35 | 0.491 | 1.51 | 0.64, 3.67 | 0.350 |
| 33 | 1.23 | 0.36, 4.48 | 0.738 | 1.14 | 0.33, 3.92 | 0.838 |
| 34 | 2.52 | 0.99, 7.03 | 0.062 | 2.70 | 1.09, 7.21 | **0.038** |
| 35 | 0.13 | 0.01, 0.75 | 0.058 | 0.16 | 0.01, 0.97 | 0.096 |
| 36 | 1.76 | 0.16, 38.8 | 0.649 | 2.27 | 0.21, 50.0 | 0.510 |
| 37 | 0.18 | 0.01, 1.16 | 0.121 | 0.23 | 0.01, 1.49 | 0.185 |
| 38 | 1.76 | 0.57, 6.09 | 0.339 | 1.70 | 0.56, 5.51 | 0.353 |
| 39 | 0.09 | 0.00, 0.49 | **0.024** | 0.11 | 0.01, 0.63 | **0.043** |
| 40 | 0.59 | 0.07, 3.73 | 0.571 | 0.76 | 0.10, 4.80 | 0.767 |
| 41 | 0.23 | 0.07, 0.64 | **0.008** | 0.23 | 0.06, 0.67 | **0.012** |
| 42 | 1.76 | 0.16, 38.8 | 0.649 | 2.27 | 0.21, 50.0 | 0.510 |
| 43 | 0.58 | 0.29, 1.15 | 0.119 | 0.55 | 0.27, 1.11 | 0.099 |
| 44 | 7.05 | 1.21, 134 | 0.072 | 3.97 | 0.89, 27.8 | 0.098 |
| 45 | 0.73 | 0.20, 2.63 | 0.632 | 0.95 | 0.25, 3.39 | 0.931 |
| 46 | 0.48 | 0.15, 1.39 | 0.187 | 0.47 | 0.14, 1.41 | 0.195 |
| 47 | 5,072,359 | 0.00, NA | 0.992 | 2,404,421 | 0.00, NA | 0.987 |
| 48 | 1.17 | 0.24, 6.29 | 0.840 | 0.85 | 0.16, 4.10 | 0.840 |
| 49 | 0.44 | 0.09, 1.79 | 0.269 | 0.57 | 0.11, 2.31 | 0.445 |
| 50 | 0.66 | 0.20, 2.07 | 0.479 | 0.45 | 0.12, 1.48 | 0.212 |
| 51 | 1.76 | 0.16, 38.8 | 0.649 | 2.27 | 0.21, 50.0 | 0.510 |
| 52 | 1.54 | 0.43, 6.28 | 0.516 | 1.99 | 0.55, 8.09 | 0.303 |
| 53 | 0.30 | 0.13, 0.65 | **0.003** | 0.31 | 0.13, 0.68 | **0.005** |
| 54 | 1.40 | 0.66, 3.00 | 0.381 | 1.80 | 0.86, 3.87 | 0.124 |
| 55 | 0.53 | 0.20, 1.33 | 0.182 | 0.47 | 0.17, 1.21 | 0.130 |
| 56 | 1.32 | 0.35, 5.51 | 0.684 | 1.70 | 0.45, 7.10 | 0.436 |
| 57 | 1.57 | 0.63, 4.10 | 0.344 | 1.23 | 0.50, 3.06 | 0.653 |
| 58 | 5,072,359 | 0.00, NA | 0.992 | 2,404,421 | 0.00, NA | 0.987 |
| 59 | 1.41 | 0.58, 3.58 | 0.457 | 1.55 | 0.64, 3.86 | 0.339 |
| 60 | 0.88 | 0.20, 3.96 | 0.864 | 1.14 | 0.25, 5.11 | 0.864 |
| 61 | 0.29 | 0.08, 0.92 | **0.048** | 0.26 | 0.06, 0.89 | **0.049** |
| 62 | 0.59 | 0.14, 2.21 | 0.436 | 0.28 | 0.04, 1.22 | 0.125 |
| 63 | 1.59 | 0.50, 5.55 | 0.443 | 2.04 | 0.65, 7.16 | 0.235 |
| 64 | 2.20 | 0.45, 16.0 | 0.362 | 1.51 | 0.31, 8.10 | 0.603 |
| **City size (reference: <5 000 inhabitants)** | | | | | | |
| 5 000 - 19 999 | 1.85 | 0.98, 3.53 | 0.059 | 2.51 | 1.32, 4.86 | **0.006** |
| 20 000 – 99 999 | 1.40 | 0.77, 2.54 | 0.273 | 1.80 | 0.98, 3.36 | 0.059 |
| ≥ 100 000 | 0.98 | 0.60, 1.63 | 0.949 | 1.23 | 0.74, 2.09 | 0.439 |
| **Height in cm** | 0.99 | 0.98, 1.00 | 0.144 | 0.99 | 0.98, 1.01 | 0.260 |
| **Weight in kg** | 1.00 | 1.0, 1.01 | 0.845 | 1.00 | 0.99, 1.01 | 0.991 |
| **Mother tongue (reference: German)** | | | | | | |
| Other | 1.72 | 1.22, 2.45 | **0.002** | 2.02 | 1.43, 2.86 | **<0.001** |
| **Self-definition as migrant (reference: No)** | | | | | | |
| Yes | 0.99 | 0.71, 1.39 | 0.973 | 1.08 | 0.76, 1.51 | 0.673 |
| **Perceived by others as migrant (reference: No)** | | | | | | |
| Yes | 1.14 | 0.83, 1.56 | 0.407 | 1.33 | 0.97, 1.82 | 0.074 |
| **Gender (reference: Female)** | | | | | | |
| Male | 0.94 | 0.73, 1.22 | 0.663 | 0.98 | 0.76, 1.28 | 0.904 |
| Diverse | 3.04 | 0.39, 61.6 | 0.336 | 4.03 | 0.51, 81.7 | 0.229 |
| **Age (years)** | 1.00 | 0.99, 1.01 | 0.564 | 0.99 | 0.98, 1.00 | 0.084 |
| **Marital status (reference: Unwed)** | | | | | | |
| Married | 0.78 | 0.57, 1.07 | 0.124 | 0.68 | 0.49, 0.94 | **0.018** |
| Divorced | 1.27 | 0.81, 2.02 | 0.301 | 0.96 | 0.61, 1.51 | 0.866 |
| Widowed | 0.80 | 0.33, 1.88 | 0.602 | 0.67 | 0.26, 1.60 | 0.377 |
| In partnership | 0.86 | 0.61, 1.22 | 0.406 | 0.88 | 0.62, 1.25 | 0.487 |
| Other | 0.95 | 0.29, 3.10 | 0.936 | 0.84 | 0.24, 2.66 | 0.765 |
| **Living situation (reference: With someone)** | | | | | | |
| Alone | 1.16 | 0.88, 1.53 | 0.280 | 1.22 | 0.93, 1.61 | 0.158 |
| **Highest schooling degree (reference: In school)** | | | | | | |
| *Sonderschulabschluss* | 4.27 | 0.81, 27.7 | 0.101 | 4.27 | 0.81, 27.7 | 0.101 |
| *Hauptschulabschluss* | 2.46 | 0.78, 8.51 | 0.131 | 1.99 | 0.63, 6.89 | 0.247 |
| *Mittlere Reife* | 2.12 | 0.69, 7.15 | 0.198 | 1.58 | 0.51, 5.33 | 0.433 |
| *Fachabitur* | 1.68 | 0.53, 5.82 | 0.386 | 1.22 | 0.38, 4.22 | 0.743 |
| *Abitur* | 1.03 | 0.34, 3.45 | 0.964 | 0.78 | 0.26, 2.62 | 0.668 |
| **Level of education (reference: No education degree)** | | | | | | |
| In education | 1.06 | 0.57, 1.96 | 0.858 | 1.02 | 0.55, 1.90 | 0.939 |
| *Lehre/Ausbildung/Fachschule* | 1.20 | 0.78, 1.83 | 0.406 | 1.07 | 0.70, 1.64 | 0.765 |
| *Meister* | 1.23 | 0.59, 2.59 | 0.575 | 0.95 | 0.45, 1.98 | 0.891 |
| University | 0.69 | 0.44, 1.09 | 0.112 | 0.60 | 0.38, 0.95 | **0.027** |
| Other | 1.63 | 0.85, 3.18 | 0.143 | 1.32 | 0.69, 2.53 | 0.401 |
| **Employment status (reference: Not in employment)** | | | | | | |
| 1 | 1.54 | 0.83, 2.87 | 0.171 | 1.53 | 0.82, 2.87 | 0.179 |
| 2 | 1.45 | 0.75, 2.83 | 0.268 | 1.77 | 0.91, 3.47 | 0.093 |
| 3 | 0.74 | 0.44, 1.27 | 0.274 | 0.75 | 0.43, 1.30 | 0.298 |
| 4 | 1.17 | 0.73, 1.90 | 0.515 | 1.25 | 0.77, 2.04 | 0.378 |
| **Smoking (reference: No)** | | | | | | |
| Yes | 1.53 | 1.18, 1.98 | **0.001** | 1.65 | 1.27, 2.14 | **<0.001** |
| **Smoking: How many units?** | 1.03 | 1.01, 1.05 | **<0.001** | 1.04 | 1.02, 1.06 | **<0.001** |
| **Previous smoking: How many cigarettes per day?** | 1.02 | 1.00, 1.03 | **0.014** | 1.01 | 1.00, 1.03 | 0.065 |
| **Years smoked** | 1.01 | 1.00, 1.02 | 0.102 | 1.01 | 1.00, 1.02 | 0.232 |
| **Alcohol drinking frequency (reference: Never)** | | | | | | |
| 1 time / month | 1.06 | 0.74, 1.52 | 0.742 | 0.96 | 0.67, 1.37 | 0.803 |
| 1-4 times / month | 0.81 | 0.56, 1.17 | 0.259 | 0.80 | 0.55, 1.16 | 0.245 |
| 1-3 times / week | 0.73 | 0.49, 1.08 | 0.119 | 0.73 | 0.49, 1.09 | 0.123 |
| ≥ 4 times / week | 1.16 | 0.65, 2.10 | 0.609 | 1.29 | 0.72, 2.31 | 0.390 |
| **Alcohol consumption: How much? (reference: *No alcohol*)** | | | | | | |
| 1-2 drinks | 0.92 | 0.67, 1.27 | 0.605 | 0.85 | 0.61, 1.17 | 0.320 |
| 3-4 drinks | 0.78 | 0.53, 1.15 | 0.203 | 0.82 | 0.55, 1.21 | 0.312 |
| 5-6 drinks | 1.16 | 0.65, 2.06 | 0.620 | 1.13 | 0.63, 2.00 | 0.684 |
| 7-8 drinks | 0.47 | 0.17, 1.18 | 0.121 | 0.60 | 0.22, 1.51 | 0.293 |
| ≥ 9 drinks | 2.60 | 0.86, 9.62 | 0.111 | 2.41 | 0.83, 7.95 | 0.120 |
| **Binge alcohol drinking (≥ 6 drinks) (reference: Never)** | | | | | | |
| ≤ 1 time / month | 1.06 | 0.76, 1.46 | 0.736 | 0.96 | 0.69, 1.34 | 0.824 |
| 1 time / month | 1.18 | 0.77, 1.82 | 0.451 | 1.13 | 0.73, 1.75 | 0.568 |
| 1 time / week | 1.02 | 0.57, 1.83 | 0.939 | 1.12 | 0.62, 2.00 | 0.710 |
| (nearly) daily | 0.77 | 0.27, 2.06 | 0.608 | 0.76 | 0.26, 2.06 | 0.602 |
| **Baseline EQ-5D-5L index** | 0.99 | 0.98, 0.99 | **<0.001** | 0.99 | 0.98, 0.99 | **<0.001** |
| **Baseline PHQ-9 score** | 1.08 | 1.04, 1.12 | **<0.001** | 1.07 | 1.03, 1.11 | **<0.001** |
| **Baseline inpatient days** | 1.01 | 0.99, 1.02 | 0.444 | 1.00 | 0.99, 1.01 | 0.885 |
| **Baseline GP visits** | 1.06 | 1.03, 1.09 | **<0.001** | 1.04 | 1.01, 1.07 | **0.007** |
| **Baseline sick leave period in the last 6 months (reference: 0 days)** | | | | | | |
| 1 week | 0.98 | 0.70, 1.37 | 0.903 | 0.93 | 0.66, 1.30 | 0.677 |
| 2-3 weeks | 1.36 | 0.97, 1.91 | 0.077 | 1.21 | 0.86, 1.70 | 0.281 |
| 1-2 month(s) | 1.34 | 0.86, 2.12 | 0.200 | 1.10 | 0.70, 1.74 | 0.670 |
| 3-4 months | 1.48 | 0.65, 3.42 | 0.348 | 1.09 | 0.47, 2.46 | 0.838 |
| > 4 months | 1.08 | 0.61, 1.91 | 0.784 | 0.71 | 0.39, 1.27 | 0.261 |
| **Baseline pharmaceutical intake** | 1.06 | 1.01, 1.11 | **0.030** | 1.05 | 1.00, 1.10 | 0.060 |
| **Risk factor (RF): Reason for GP visit (reference: physical symptoms)** | | | | | | |
| Mental symptoms | 0.97 | 0.70, 1.34 | 0.857 | 0.90 | 0.65, 1.25 | 0.541 |
| Neither | 0.71 | 0.51, 1.00 | 0.051 | 0.75 | 0.53, 1.05 | 0.101 |
| **RF: Anxieties (reference: No)** | | | | | | |
| Yes | 1.21 | 0.94, 1.56 | 0.142 | 1.24 | 0.96, 1.61 | 0.095 |
| **RF: Addiction (reference: No)** | | | | | | |
| Yes | 1.53 | 1.17, 2.01 | **0.002** | 1.63 | 1.25, 2.15 | **<0.001** |
| **RF: Traumatic event (reference: No)** | | | | | | |
| Yes | 1.05 | 0.80, 1.37 | 0.735 | 1.04 | 0.80, 1.37 | 0.759 |
| **RF: Persistent somatic symptoms (reference: No)** | | | | | | |
| Yes | 1.42 | 1.10, 1.84 | **0.008** | 1.37 | 1.06, 1.79 | **0.018** |
| **RF: Mood swings (reference: No)** | | | | | | |
| Yes | 0.80 | 0.52, 1.23 | 0.312 | 0.72 | 0.47, 1.11 | 0.134 |
| **RF: Chronic physical disease (reference: No)** | | | | | | |
| Yes | 0.98 | 0.76, 1.26 | 0.859 | 0.89 | 0.68, 1.15 | 0.367 |
| **RF: Social support (reference: No)** | | | | | | |
| Yes | 0.62 | 0.47, 0.82 | **0.001** | 0.59 | 0.44, 0.78 | **<0.001** |
| **Previous depression diagnosis (reference: Never)** | | | | | | |
| 1-5 years ago | 0.81 | 0.58, 1.14 | 0.226 | 0.72 | 0.51, 1.01 | 0.061 |
| 5-10 years ago | 1.26 | 0.83, 1.94 | 0.284 | 1.02 | 0.66, 1.55 | 0.939 |
| > 10 years ago | 0.89 | 0.54, 1.48 | 0.665 | 1.00 | 0.59, 1.66 | 0.989 |
| **Previous depression treatment (reference: Never)** | | | | | | |
| 1-5 years ago | 0.71 | 0.51, 0.99 | **0.044** | 0.68 | 0.48, 0.95 | **0.024** |
| 5-10 years ago | 1.44 | 0.91, 2.29 | 0.124 | 1.13 | 0.72, 1.78 | 0.591 |
| > 10 years ago | 0.57 | 0.28, 1.11 | 0.103 | 0.65 | 0.32, 1.27 | 0.216 |
| **Treatment type (reference: *No treatment*)** | | | | | | |
| Psychotherapy | 0.94 | 0.64, 1.40 | 0.770 | 0.78 | 0.52, 1.17 | 0.235 |
| Antidepressants | 0.79 | 0.41, 1.49 | 0.467 | 0.81 | 0.42, 1.54 | 0.532 |
| PT + AD | 0.84 | 0.58, 1.19 | 0.328 | 0.80 | 0.55, 1.15 | 0.233 |
| **Diagnosis: Who? (reference: *No diagnosis*)** | | | | | | |
| GP | 0.93 | 0.65, 1.33 | 0.699 | 0.88 | 0.61, 1.25 | 0.468 |
| Psychotherapist | 0.89 | 0.56, 1.42 | 0.628 | 0.83 | 0.51, 1.32 | 0.439 |
| Psychiatrist | 1.04 | 0.61, 1.79 | 0.874 | 1.00 | 0.58, 1.71 | 0.996 |
| Neurologist | 1.46 | 0.62, 3.58 | 0.392 | 0.88 | 0.36, 2.07 | 0.772 |
| Other physician | 0.59 | 0.22, 1.48 | 0.272 | 0.45 | 0.15, 1.20 | 0.134 |
| **RF: Other mental disorder (reference: Never)** | | | | | | |
| In the last year | 0.77 | 0.45, 1.28 | 0.315 | 0.70 | 0.40, 1.19 | 0.195 |
| 1-5 years ago | 1.01 | 0.66, 1.54 | 0.962 | 1.01 | 0.66, 1.54 | 0.963 |
| 5-10 years ago | 1.43 | 0.81, 2.56 | 0.215 | 1.24 | 0.70, 2.18 | 0.455 |
| > 10 years ago | 1.28 | 0.72, 2.28 | 0.396 | 1.02 | 0.57, 1.80 | 0.958 |
| **RF: Mental disorder in the family (reference: No)** | | | | | | |
| Yes | 0.93 | 0.72, 1.20 | 0.568 | 0.87 | 0.67, 1.12 | 0.275 |
| **RF: Suicidal ideation in the family (reference: No)** | | | | | | |
| Yes | 0.98 | 0.73, 1.32 | 0.917 | 0.98 | 0.72, 1.31 | 0.869 |
| **RF: Pregnancy planned (reference: No)** | | | | | | |
| Yes | 0.96 | 0.44, 2.08 | 0.917 | 0.79 | 0.35, 1.72 | 0.561 |
| **RF: Currently pregnant (reference: No)** | | | | | | |
| Yes | 0.51 | 0.07, 2.65 | 0.444 | 0.67 | 0.09, 3.47 | 0.649 |
| **RF: Gave birth in the last 6 months (reference: No)** | | | | | | |
| Yes | 2.07 | 0.20, 44.7 | 0.552 | 2.71 | 0.26, 58.5 | 0.416 |
| **RF: Currently breastfeeding (reference: No)** | | | | | | |
| Yes | 0.52 | 0.07, 2.65 | 0.445 | 0.67 | 0.09, 3.47 | 0.650 |
| **RF: PMS (reference: No)** | | | | | | |
| Yes | 1.09 | 0.78, 1.53 | 0.617 | 1.15 | 0.82, 1.61 | 0.414 |
| **RF: Menopausal (reference: No)** | | | | | | |
| Yes | 0.92 | 0.64, 1.33 | 0.666 | 0.70 | 0.47, 1.02 | 0.069 |
| **Study center (reference: HD)** | | | | | | |
| **HH** | 0.68 | 0.31, 1.46 | 0.315 | 0.56 | 0.26, 1.22 | 0.137 |
| **JE** | 1.46 | 0.66, 3.26 | 0.348 | 1.37 | 0.62, 3.07 | 0.435 |
| **MU** | 1.32 | 0.60, 2.92 | 0.485 | 1.19 | 0.54, 2.66 | 0.658 |
| **TÜ** | 1.20 | 0.49, 2.93 | 0.686 | 1.09 | 0.45, 2.66 | 0.855 |
| **Feedback group (reference: No feedback)** | | | | | | |
| GP-feedback | 1.12 | 0.82, 1.52 | 0.483 | 1.06 | 0.78, 1.45 | 0.694 |
| GP-patient-feedback | 0.90 | 0.66, 1.22 | 0.483 | 0.95 | 0.70, 1.30 | 0.752 |

1. Visual representation of missings per target variable of MI


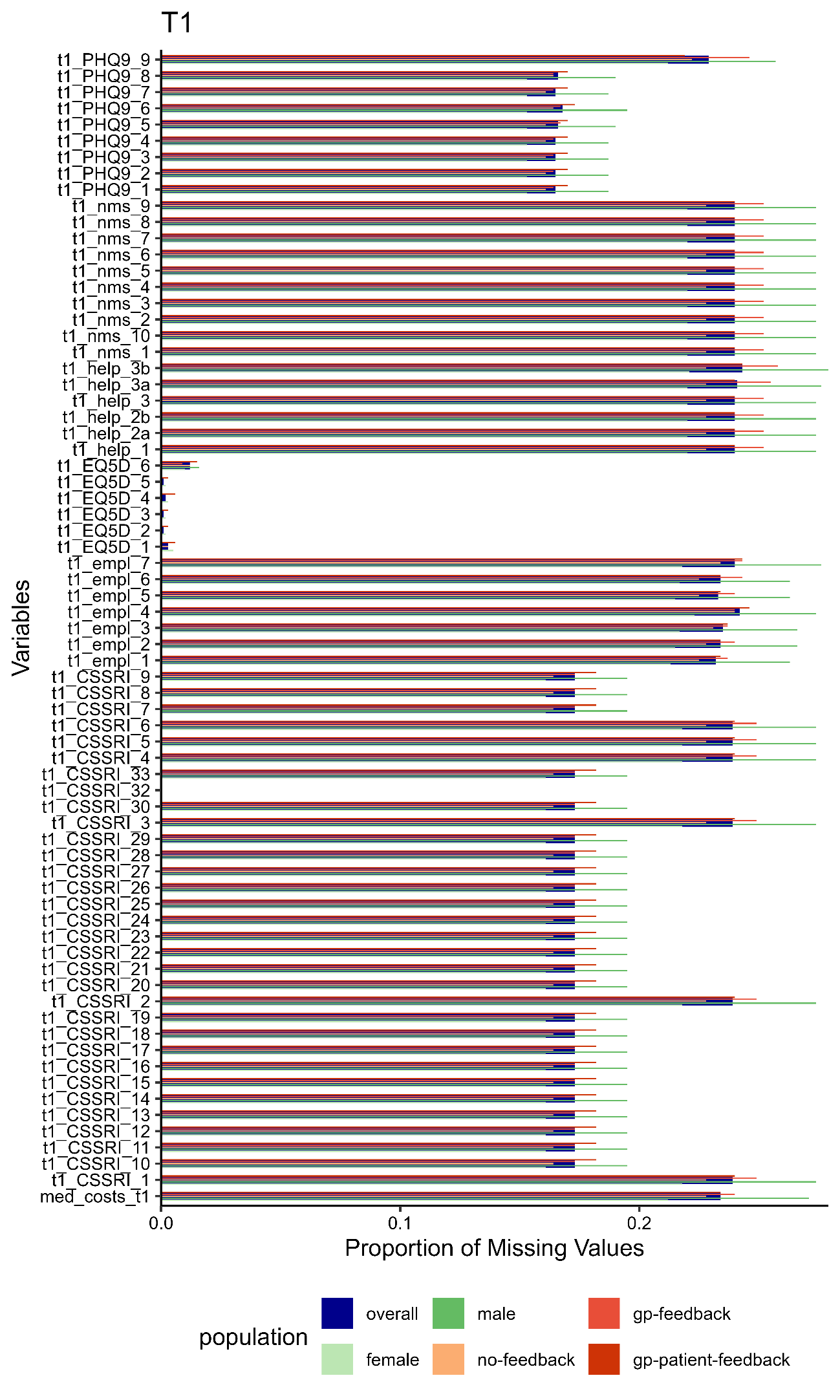


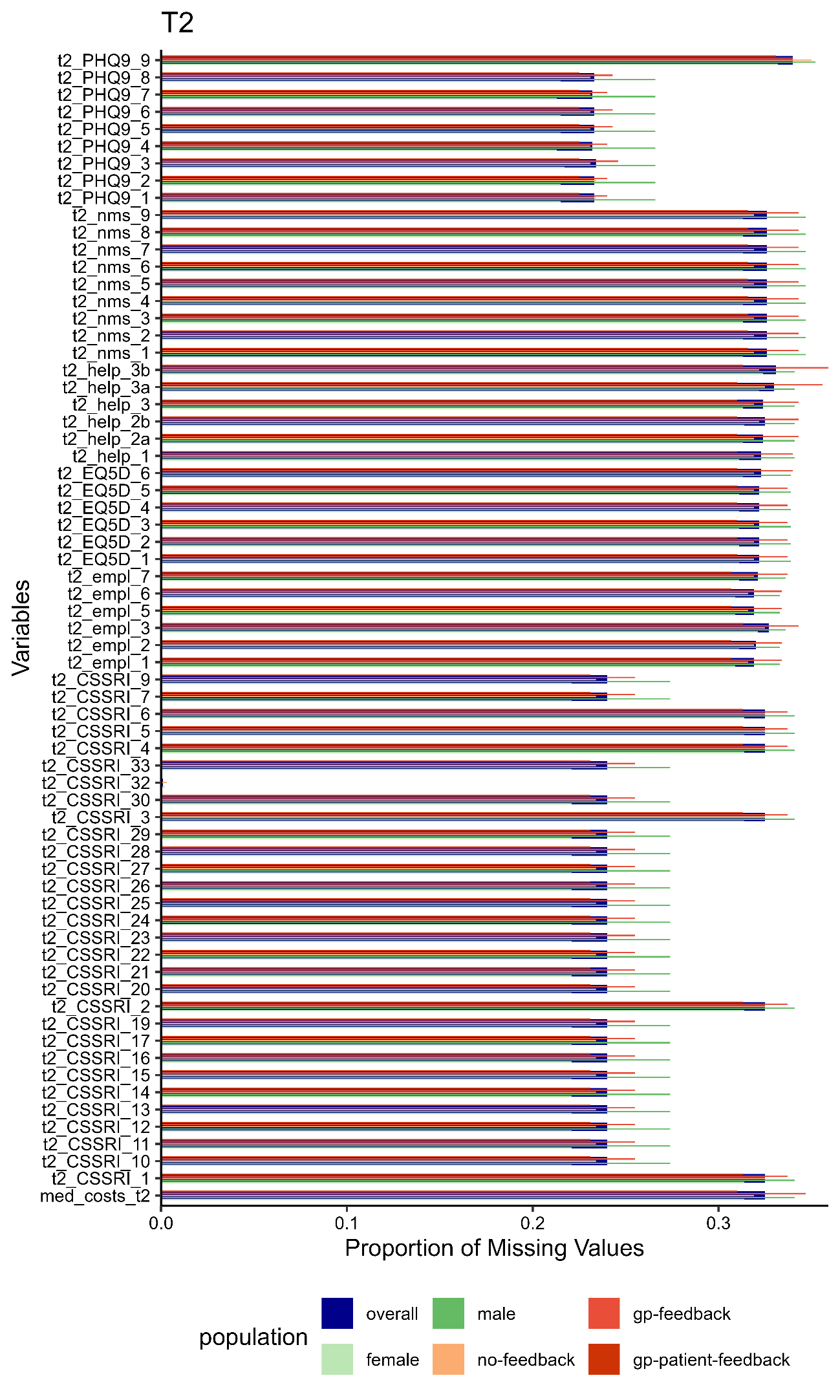


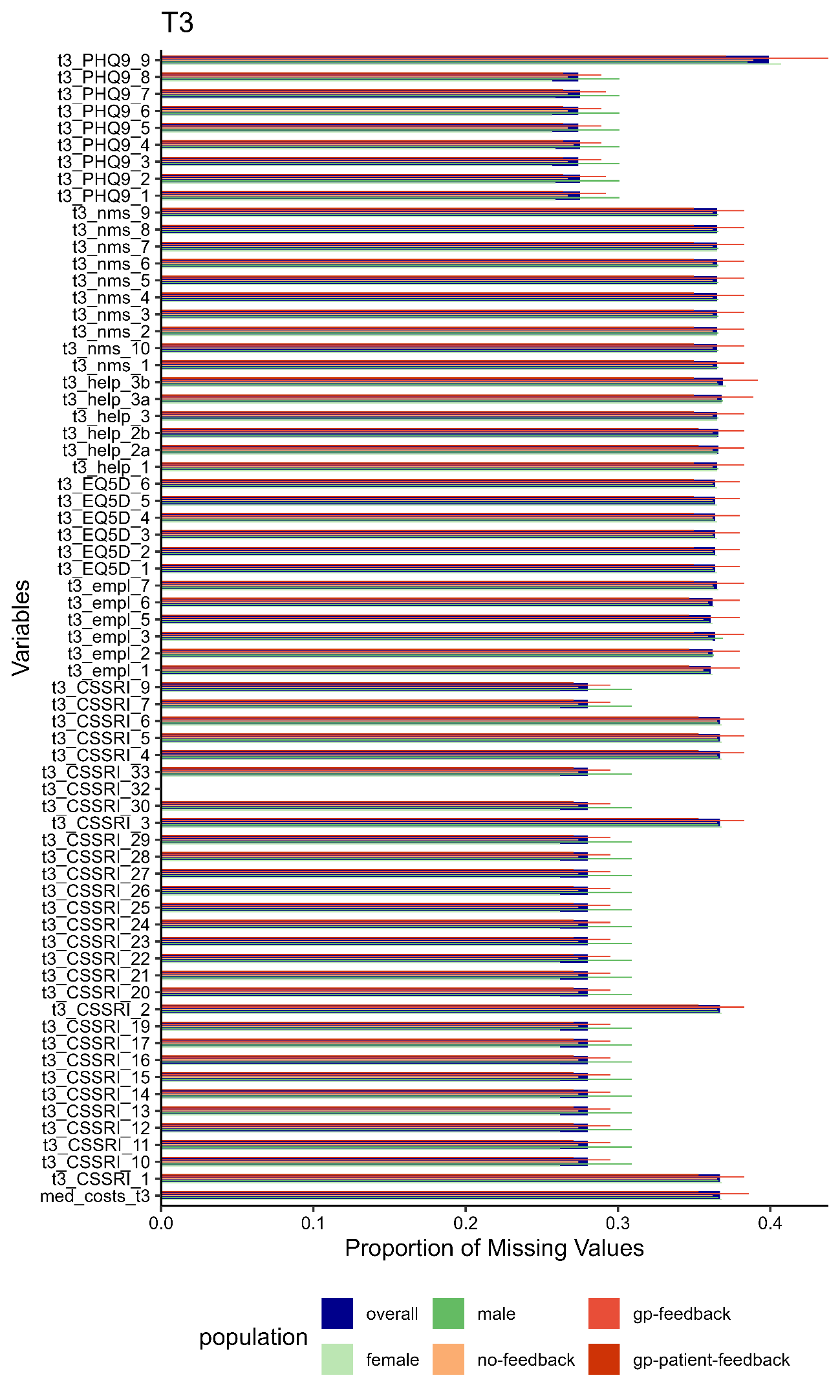

Supplement: Kreis et al. supplementary material 5 — Kreis et al. supplementary material [file S2056472425109459sup005.docx]
