# Supplementary material for: Cost-effectiveness of targeted feedback interventions after depression screening in primary care: health economic evaluation of the GET.FEEDBACK.GP trial
Source: BJPsych Open. 2026 Feb 2;12(2):e52. doi: 10.1192/bjo.2025.10945 (PMC12926889; doi:10.1192/bjo.2025.10945)
Supplement: Kreis et al. supplementary material 6 — Kreis et al. supplementary material [file S2056472425109459sup006.docx]

**Supplementary Material 6:**

Unadjusted costs and HRQL during follow-up (12 months):

| Category | | No feedback group | GP-targeted feedback group | GP-targeted plus patient-targeted feedback group |
| --- | --- | --- | --- | --- |
|  | | Mean (SE) | Mean (SE) | Mean (SE) |
| Direct costs [€] | | 7093 (1493) | 8095 (1532) | 7852 (1319) |
|  | Inpatient services [€] | 4113 (1481) | 4260 (1568) | 4293 (1324) |
|  | Outpatient physician services (w/o psychotherapy) [€] | 906 (61) | 893 (63) | 878 (63) |
|  | Outpatient psychiatric and psychotherapy services [€] | 405 (70) | 609 (71) | 541 (70) |
|  | Outpatient non-physician services [€] | 374 (65) | 473 (66) | 395 (61) |
|  | Formal nursing care [€] | 59 (56) | 86 (59) | 143 (54) |
|  | Informal care [€] | 794 (264) | 993 (267) | 839 (253) |
|  | Medication [€] | 443 (197) | 781 (181) | 764 (198) |
| Indirect costs [€] | | 5846 (779) | 6176 (889) | 5364 (841) |
| Total costs [€] | | 12 939 (1798) | 14 271 (1852) | 13 216 (1634) |
| EQ-5D-5L index (baseline) | | 0.66 (0.01) | 0.66 (0.01) | 0.71 (0.01) |
| EQ-5D-5L index (1 month follow-up) | | 0.66 (0.01) | 0.66 (0.01) | 0.71 (0.01) |
| EQ-5D-5L index (6 months follow-up) | | 0.77 (0.03) | 0.78 (0.03) | 0.81 (0.03) |
| EQ-5D-5L index (12 months follow-up) | | 0.81 (0.01) | 0.79 (0.01) | 0.81 (0.01) |
| PHQ-9 score (baseline) | | 13.6 (0.2) | 13.4 (0.2) | 13.5 (0.2) |
| PHQ-9 score (1 month follow-up) | | 9.9 (0.3) | 10.3 (0.3) | 10.1 (0.3) |
| PHQ-9 score (6 months follow-up) | | 9.3 (0.3) | 9.3 (0.3) | 8.9 (0.3) |
| PHQ-9 score (12 months follow-up) | | 8.7 (0.3) | 8.9 (0.3) | 8.6 (0.3) |
