# Supplementary material for: Cost-effectiveness of targeted feedback interventions after depression screening in primary care: health economic evaluation of the GET.FEEDBACK.GP trial
Source: BJPsych Open. 2026 Feb 2;12(2):e52. doi: 10.1192/bjo.2025.10945 (PMC12926889; doi:10.1192/bjo.2025.10945)
Supplement: Kreis et al. supplementary material 7 — Kreis et al. supplementary material [file S2056472425109459sup007.docx]

**Supplementary Material 7:**

Resource use per study arm during follow-up (12 months):

| Category | No feedback group | GP-targeted feedback group | GP-targeted plus patient-targeted feedback group |
| --- | --- | --- | --- |
|  | Mean | Mean | Mean |
| ***Inpatient stays (number of days)*** | | | |
| General hospital | 2.1 | 1.9 | 2.0 |
| Day-care general hospital | 0.4 | 0.1 | 0.3 |
| Psychiatric hospital | 1.1 | 1.5 | 1.8 |
| Day-care psychiatric hospital | 1.8 | 2.6 | 2.2 |
| Rehabilitation clinic | 1.5 | 1.6 | 1.0 |
| Day-care rehabilitation clinic | 0.1 | 0.4 | 0.2 |
| ***Outpatient physician contacts (number of visits)*** | | | |
| General practitioner | 7.1 | 7.1 | 6.6 |
| Internist | 0.1 | 0.2 | 0.1 |
| Cardiologist | 0.3 | 0.2 | 0.2 |
| Pulmonologist | 0.2 | 0.3 | 0.2 |
| Endocrinologist | 0.1 | 0.1 | 0.1 |
| Diabetologist | 0.1 | 0.1 | 0.1 |
| Nephrologist | 0.0 | 0.1 | 0.0 |
| Orthopedist | 1.3 | 1.4 | 1.1 |
| Psychiatrist | 0.7 | 0.9 | 0.6 |
| Neurologist | 0.4 | 0.4 | 0.3 |
| Dermatologist | 0.5 | 0.5 | 0.5 |
| Ear-nose-throat (ENT) specialist | 0.6 | 0.6 | 0.6 |
| Outpatient surgeon | 0.2 | 0.3 | 0.3 |
| Radiologist | 0.6 | 0.5 | 0.4 |
| Urologist | 0.2 | 0.2 | 0.2 |
| Ophthalmologist | 0.5 | 0.3 | 0.3 |
| Gynaecologist | 0.9 | 0.7 | 0.9 |
| Rheumatologist | 0.1 | 0.1 | 0.0 |
| Dentist / Orthodentist | 1.6 | 2.4 | 2.1 |
| Emergency physician | 0.1 | 0.1 | 0.1 |
| Hospital outpatient clinic | 0.2 | 0.2 | 0.2 |
| Emergency service (116 117) | 0.1 | 0.1 | 0.1 |
| Further specialist | 0.2 | 0.3 | 0.4 |
| Psychologist / Psychotherapist | 3.1 | 4.9 | 4.4 |
| ***Non-medical services contacts (number of visits)*** | | | |
| Ergotherapy | 1.2 | 1.8 | 1.3 |
| Alternative practitioner | 0.5 | 0.5 | 0.5 |
| Inhalation therapy | 0.0 | 0.1 | 0.1 |
| Physiotherapy | 6.3 | 6.8 | 5.7 |
| Massage or lymphatic drainage | 1.8 | 2.1 | 1.9 |
| Sports or exercise therapy | 1.2 | 1.5 | 1.2 |
| Speech therapy | 0.2 | 0.4 | 0.5 |
| Heat, cold and water therapy | 0.3 | 0.8 | 0.7 |
| Self-help groups | 0.6 | 1.0 | 0.7 |
| Further non-medical services | 0.6 | 0.5 | 0.4 |
| ***Care (hours)*** | | | |
| Professional care | 1.4 | 2.1 | 3.7 |
| Informal care | 27.2 | 34.1 | 28.8 |
| ***Absenteeism*** | | | |
| Sick leave (days) | 27.3 | 28.9 | 24.9 |
| Leaves due to medical or therapeutic contacts (hours) | 5.7 | 5.4 | 5.8 |
