# Supplementary material for: Cost-effectiveness of targeted feedback interventions after depression screening in primary care: health economic evaluation of the GET.FEEDBACK.GP trial
Source: BJPsych Open. 2026 Feb 2;12(2):e52. doi: 10.1192/bjo.2025.10945 (PMC12926889; doi:10.1192/bjo.2025.10945)
Supplement: Kreis et al. supplementary material 8 — Kreis et al. supplementary material [file S2056472425109459sup008.docx]

**Supplementary Material 8: Results of subpopulation analyses**

| **Category** | **GP-targeted feedback** compared to **no feedback** | **GP-targeted plus patient-targeted feedback** compared to **no feedback** |
| --- | --- | --- |
|  | **Adjusted differences (∆) between groups^1^ [95%-CI]** | |
| **Female: 614/987 (62%)**   - **No feedback: 211/614 (34%)** - **GP-targeted feedback: 205/614 (33%)** - **GP-targeted plus patient-targeted feedback: 198/614 (32%)** | | |
| ***Societal perspective (base case)*** | | |
| Total costs to society [€] | +50 [-1197; +1630] | +70 [-1311; +1871] |
| QALY | -0.006 [-0.030; +0.018] | +0.006 [-0.019; +0.030] |
| ***Payer perspective*** | | |
| Total payer costs [€] | +66 [-760; 1162] | +175 [-649; +1255] |
| QALY | -0.006 [-0.030; +0.018] | +0.007 [-0.017; +0.031] |
| ***Complete case analysis*** | | |
| Total costs to society [€] | **-1478 [-1505; -1450]** | **-226 [-260; -191]** |
| QALY | +0.012 [-0.018; +0.042] | +0.021 [-0.008; +0.050] |
| ***Depression-free days (DFD) as effect measure*** | | |
| Total costs to society [€] | +50 [-1197; +1630] | +70 [-1311; +1871] |
| DFD | -6.6 [-24.0; +10.9] | +0.5 [-17.5; +18.5] |
| **Male: 369/987 (37%)**   - **No feedback: 117/369 (32%)** - **GP-targeted feedback: 124/369 (34%)** - **GP-targeted plus patient-targeted feedback: 128/369 (35%)** | | |
| ***Societal perspective (base case)*** | | |
| Total costs to society [€] | +1870 [-498; +5062] | +1600 [-624; +4565] |
| QALY | +0.003 [-0.025; +0.032] | -0.006 [-0.035: +0.024] |
| ***Payer perspective*** | | |
| Total payer costs [€] | +1765 [-155; +4769] | **+1960 [+321; +4293]** |
| QALY | +0.007 [-0.022; +0.035] | -0.003 [-0.033; +0.027] |
| ***Complete case analysis*** | | |
| Total costs to society [€] | +106 [-232; +628] | +115 [-219; +626] |
| QALY | -0.004 [-0.040; +0.033] | -0.004 [-0.040; 0.031] |
| ***Depression-free days (DFD) as effect measure*** | | |
| Total costs to society [€] | +1870 [-498; +5062] | +1600 [-624; +4565] |
| DFD | -6.3 [-29.3; +16.7] | -14.0 [-37.2; +9.1] |

1. Gender (female/male)
2. Depression history (yes/no)

| **Category** | **GP-targeted feedback** compared to **no feedback** | **GP-targeted plus patient-targeted feedback** compared to **no feedback** |
| --- | --- | --- |
|  | **Adjusted differences (∆) between groups^1^ [95%-CI]** | |
| **With previous depression: 342/987 (35%)**   - **No feedback: 111/342 (32%)** - **GP-targeted feedback: 117/342 (34%)** - **GP-targeted plus patient-targeted feedback: 114/342 (33%)** | | |
| ***Societal perspective (base case)*** | | |
| Total costs to society [€] | +641 [-515; +2234] | -249 [-1117; +925] |
| QALY | -0.009 [-0.046; +0.028] | +0.023 [-0.013; +0.059] |
| ***Payer perspective*** | | |
| Total payer costs [€] | +941 [-144; +2516] | +96 [-658; +1150] |
| QALY | -0.012 [-0.048; +0.025] | +0.023 [-0.013; +0.059] |
| ***Complete case analysis*** | | |
| Total costs to society [€] | -225 [-877; +659] | **-455 [-466; -443]** |
| QALY | -0.003 [-0.050; +0.044] | +0.031 [-0.015; +0.078] |
| ***Depression-free days (DFD) as effect measure*** | | |
| Total costs to society [€] | +641 [-515; +2234] | -249 [-1117; +925] |
| DFD | -15.5 [-38.5; +7.5] | +8.8 [-14.7; +32.3] |
| **Without previous depression: 645/987 (65%)**   - **No feedback: 218/645 (34%)** - **GP-targeted feedback: 212/645 (33%)** - **GP-targeted plus patient-targeted feedback: 215/645 (33%)** | | |
| ***Societal perspective (base case)*** | | |
| Total costs to society [€] | +220 [-1640; +2545] | +1259 [-771; +3785] |
| QALY | +0.004 [-0.015; +0.024] | -0.004 [-0.024; +0.016] |
| ***Payer perspective*** | | |
| Total payer costs [€] | +267 [-785; +1676] | +1129 [-238; +3006] |
| QALY | +0.005 [-0.015; +0.025] | -0.005 [-0.025; +0.016] |
| ***Complete case analysis*** | | |
| Total costs to society [€] | **-121 [-166; -76]** | **+673 [+618; +728]** |
| QALY | +0.008 [-0.017; +0.033] | +0.000 [-0.024; +0.025] |
| ***Depression-free days (DFD) as effect measure*** | | |
| Total costs to society [€] | +220 [-1640; +2545] | +1259 [-771; +3785] |
| DFD | +0.4 [-16.9; +17.6] | -7.0 [-24.3; +10.2] |

1. Any behavioral or substance addiction (yes/no)

| **Category** | **GP-targeted feedback** compared to **no feedback** | **GP-targeted plus patient-targeted feedback** compared to **no feedback** |
| --- | --- | --- |
|  | **Adjusted differences (∆) between groups^1^ [95%-CI]** | |
| **With substance addiction: 308/987 (31%)**   - **No feedback: 103/308 (33%)** - **GP-targeted feedback: 109/308 (35%)** - **GP-targeted plus patient-targeted feedback: 96/308 (31%)** | | |
| ***Societal perspective (base case)*** | | |
| Total costs to society [€] | +92 [-2869; +4154] | +356 [-3046; +5244] |
| QALY | -0.005 [-0.038; +0.028] | -0.009 [-0.046; +0.028] |
| ***Payer perspective*** | | |
| Total payer costs [€] | +147 [-1580; +2804] | +664 [-1328; +3803] |
| QALY | -0.004 [-0.037; +0.029] | -0.008 [-0.045; +0.029] |
| ***Complete case analysis*** | | |
| Total costs to society [€] | +59 [-1824; +2913] | -582 [-2270; +1994] |
| QALY | +0.005 [-0.045; +0.055] | +0.005 [-0.047; +0.056] |
| ***Depression-free days (DFD) as effect measure*** | | |
| Total costs to society [€] | +92 [-2869; +4154] | +356 [-3046; +5244] |
| DFD | -15.1 [-39.5; +9.4] | -22.6 [-48.9; +3.7] |
| **Without any substance addiction: 679/987 (69%)**   - **No feedback: 226/679 (33%)** - **GP-targeted feedback: 220/679 (32%)** - **GP-targeted plus patient-targeted feedback: 233/679 (34%)** | | |
| ***Societal perspective (base case)*** | | |
| Total costs to society [€] | +524 [-658; +2063] | +258 [-770; +1566] |
| QALY | +0.000 [-0.021; +0.022] | +0.006 [-0.016; +0.028] |
| ***Payer perspective*** | | |
| Total payer costs [€] | +468 [-136; +1263] | +524 [-89; +1329] |
| QALY | +0.000 [-0.021; +0.022] | +0.009 [-0.014; +0.031] |
| ***Complete case analysis*** | | |
| Total costs to society [€] | -140 [-1143; +1206] | +675 [-493; +2224] |
| QALY | +0.009 [-0.017; +0.035] | +0.012 [-0.013; +0.037] |
| ***Depression-free days (DFD) as effect measure*** | | |
| Total costs to society [€] | +524 [-658; +2063] | +258 [-770; +1566] |
| DFD | -2.5 [-19.3; +14.3] | +1.9 [-15.1; +18.9] |

1. Diagnosis (1 month after baseline via the MINI diagnostic interview)

| **Category** | **GP-targeted feedback** compared to **no feedback** | **GP-targeted plus patient-targeted feedback** compared to **no feedback** |
| --- | --- | --- |
|  | **Adjusted differences (∆) between groups^1^ [95%-CI]** | |
| **Diagnosed: 279/751 (37%)**   - **No feedback: 94/279 (34%)** - **GP-targeted feedback: 99/279 (35%)** - **GP-targeted plus patient-targeted feedback: 86/279 (31%)** | | |
| ***Societal perspective (base case)*** | | |
| Total costs to society [€] | +255 [-3122; +5069] | -2200 [-4991; +1875] |
| QALY | +0.018 [-0.019; +0.056] | +0.030 [-0.010; +0.070] |
| ***Payer perspective*** | | |
| Total payer costs [€] | +48 [-2306; +3678] | -783 [-3114; +3083] |
| QALY | +0.018 [-0.020; +0.055] | +0.031 [-0.009; +0.070] |
| ***Complete case analysis*** | | |
| Total costs to society [€] | -2573 [-4758; +609] | -2293 [-4632; +1155] |
| QALY | +0.026 [-0.020; +0.072] | +0.047 [-0.000; +0.095] |
| ***Depression-free days (DFD) as effect measure*** | | |
| Total costs to society [€] | +255 [-3122; +5069] | -2200 [-4991; +1875] |
| DFD | -6.9 [-31.1; +17.4] | +2.9 [-22.5; +28.4] |
| **Not diagnosed: 472/751 (63%)**   - **No feedback: 160/472 (34%)** - **GP-targeted feedback: 149/472 (32%)** - **GP-targeted plus patient-targeted feedback: 163/472 (34%)** | | |
| ***Societal perspective (base case)*** | | |
| Total costs to society [€] | +635 [-495; +2134] | +1356 [-70; +3305] |
| QALY | -0.012 [-0.033; +0.010] | -0.002 [-0.023; +0.020] |
| ***Payer perspective*** | | |
| Total payer costs [€] | +369 [-343; +1401] | +1227 [-22; +3294] |
| QALY | -0.011 [-0.032; +0.011] | -0.001 [-0.023; +0.020] |
| ***Complete case analysis*** | | |
| Total costs to society [€] | +294 [-299; +1074] | **+856 [+148; +1777]** |
| QALY | -0.007 [-0.031; +0.018] | -0.003 [-0.026; +0.020] |
| ***Depression-free days (DFD) as effect measure*** | | |
| Total costs to society [€] | +635 [-495; +2134] | +1356 [-70; +3305] |
| DFD | +0.6 [-18.1; +19.3] | +2.3 [-16.0; +20.6] |
| Note: 236 of the 987 participants (24%) did not complete the MINI diagnosis criterion. However, in this analysis, complete multiply imputed datasets were used, which had the same pooled proportions of diagnosed and not-diagnosed participants. | | |

**Overall remarks for subpopulation analyses:**

^1^ Differences in costs were estimated using generalised linear mixed effects models assuming a Gamma distribution and a log-link function. Effect differences in QALYs and DFDs were estimated using linear mixed effects regression models. All values are reported for the 12-months follow-up period.

DFD: Depression-free days

QALY: Quality-adjusted life years

All costs are reported as 2022-€
